# Exogenous Auxin Elicits Changes in the *Arabidopsis thaliana* Root Proteome in a Time-Dependent Manner

**DOI:** 10.3390/proteomes5030016

**Published:** 2017-07-10

**Authors:** William O. Slade, W. Keith Ray, Sherry B. Hildreth, Brenda S. J. Winkel, Richard F. Helm

**Affiliations:** 1 Department of Biological Sciences, Virginia Tech, Blacksburg, VA 24061, USA; woslade@gmail.com (W.O.S.); shildret@vt.edu (S.B.H.); winkel@vt.edu (B.S.J.W.); 2 Department of Biochemistry, Virginia Tech, Blacksburg, VA 24061, USA; wkray@vt.edu

**Keywords:** auxin, label-free, root, time course

## Abstract

Auxin is involved in many aspects of root development and physiology, including the formation of lateral roots. Improving our understanding of how the auxin response is mediated at the protein level over time can aid in developing a more complete molecular framework of the process. This study evaluates the effects of exogenous auxin treatment on the *Arabidopsis* root proteome after exposure of young seedlings to auxin for 8, 12, and 24 h, a timeframe permitting the initiation and full maturation of individual lateral roots. Root protein extracts were processed to peptides, fractionated using off-line strong-cation exchange, and analyzed using ultra-performance liquid chromatography and data independent acquisition-based mass spectrometry. Protein abundances were then tabulated using label-free techniques and evaluated for significant changes. Approximately 2000 proteins were identified during the time course experiment, with the number of differences between the treated and control roots increasing over the 24 h time period, with more proteins found at higher abundance with exposure to auxin than at reduced abundance. Although the proteins identified and changing in levels at each time point represented similar biological processes, each time point represented a distinct snapshot of the response. Auxin coordinately regulates many physiological events in roots and does so by influencing the accumulation and loss of distinct proteins in a time-dependent manner. Data are available via ProteomeXchange with the identifier PXD001400.

## 1. Introduction

Auxin regulates many critical aspects of root development and physiology, including the response to gravity and light [1], the orientation and extent of cell division [2], and the initiation of lateral roots [3]. These processes are controlled by the establishment and maintenance of distinct intracellular pools of auxin generated by cellular- and tissue-specific gradients of the hormone [2]. While the actual concentration of free auxin is low relative to conjugates, the establishment of auxin gradients leads to transcriptional regulation of genes and changes in proteome profiles and enzyme activities, ultimately leading to alteration of root physiology. 

The present working model of lateral root formation consists of programed cell death processes in distal lateral root cap cells leading to the cyclical release of auxin. The transport of this released auxin to pericycle cells leads to the conversion of a subset of these cells to lateral root primordia and subsequently lateral roots. The biochemistry of lateral root formation beyond these initial cell fate decisions suggests a suite of processes both inside (symplastic) and outside (apoplastic) the plasma membrane [4]. 

The effects of auxin on *Arabidopsis* root growth at the protein level has been reviewed [5]. Zhang et al. [6] observed changes in the phosphorylation status of 20 proteins, including SORTING NEXIN 1 (SNX1), after exposure of roots to auxin for 2 h. The additional mutational analysis of serine 16 of SNX1 demonstrated its involvement in lateral root formation. In previous work from our laboratory using a 2D gel approach, auxin (indoleacetic acid, IAA) treatment increased the abundance of adenosine kinase 2 (ADK2) and rhamnose biosynthesis 1 (RHM1), both of which have previously been associated with pectin development [7].

Shotgun or untargeted LC-MS approaches to protein identification have traditionally relied upon data-dependent acquisition (DDA) methods, where peptide ions are selected for fragmentation based on user-established intensity thresholds. Although DDA is popular due to its ease of use and high throughput, it is limited by the dynamic range of complex protein samples and the stochastic nature of molecular ion sampling in a mass spectrometer duty cycle [8]. In contrast, data-independent acquisition (DIA) fragments molecular ions simultaneously with data processing protocols used to align precursor ions to their corresponding fragment ions [9]. Researchers have used this methodology to achieve increased accuracy of quantitation and increased dynamic range over DDA-based methods [10,11,12].

Herein we describe the effects of exogenous auxin on the *Arabidopsis thaliana* root proteome as observed using UPLC and DIA-MS. *Arabidopsis* seedlings were exposed to IAA for 8, 12, or 24 h using the same protocol described in recent transcriptome profiling experiments [13,14], in order to evaluate the correlation between the changes in transcript levels and those observed at the level of the proteome. Although proteins with similar cellular functions were identified as being affected by auxin at each time point, there was little overlap with respect to the specific proteins exhibiting altered abundance between the three time points. This is in contrast to what was observed at the transcript level, where the same sets of genes appear to be coordinately regulated over multiple time points [13]. Moreover, there was no direct correlation between changes in transcript and protein abundances within time-matched samples.

## 2. Materials and Methods 

### 2.1. Plant Material and Hormone Treatment

Plant material was prepared as described previously [7]. Briefly, seeds of the Columbia (Col-0) ecotype of *Arabidopsis* were obtained from Lehle Seeds (Round Rock, TX, USA) and grown on 1x Murashige and Skoog media from Caisson Labs (North Logan, UT, USA) pH 5.7, and 0.8% (*w*/*v*) agar supplemented with 1% (*w*/*v*) sucrose. After cold treatment for 48–72 h at 4 °C, seeds were moved to a growth chamber and grown under 100 μmol m^−2^·s^−2^ cool-white light (24 h lighting). The growth chamber was maintained at 22 °C and 60% relative humidity. All assays were conducted 5 d after germination, which was typically 6–7 d after cold treatment. Hormone-transfer assays were conducted as described previously [14]. Plants were germinated on nylon mesh (03-100/32; Sefar Filtration, Depew, NY, USA) pressed tightly against control media with approximately 200 seedlings per plate. At 5 d post-germination, the filter was transferred to a control plate or a plate containing media supplemented with 1 μM IAA. After 8, 12, or 24 h, seedlings were collected and immediately frozen in liquid N_2_. A razor blade was then used to carefully excise the roots at the root-shoot junction. Roots were stored at −80 °C until protein extraction. 

### 2.2. Protein Extraction and Processing

Each biological replicate (~150 mg) was ground to a fine powder and mixed with three volumes of extraction buffer (100 mM Tris-HCl, (tris(hydroxyamino)methane hydrochloride)) at pH 8.0 that was supplemented with 2% SDS (sodium dodecylsulfate, (*w*/*v*)), 1% β-mercaptoethanol (*v*/*v*), 5 mM EGTA (triethylene glycol diamine tetraacetic acid), and 10 mM EDTA (ethylenediamine tetraacetic acid). The mixture was heated for 10 min at 65 °C, and then centrifuged (6710× *g*, 20 min). The supernatant was mixed with one volume of ice-cold Tris-buffered phenol (pH 7.5), and centrifuged (6710× *g*, 6 min) to separate the aqueous and phenol phases. The aqueous phase was removed leaving the interface intact, and the phenol phase was extracted twice with ice-cold 50 mM Tris-HCl (pH 8.0). The final phenol phase was mixed with five volumes of ice-cold 0.1 M (NH_4_)OAc in methanol (MeOH). Precipitated protein was pelleted by centrifugation and the supernatant discarded. The pellet was washed once with ethanol then solubilized in resuspension buffer (7 M urea, 2 M thiourea, and 4% CHAPS (3-[(3-cholamidopropyl)dimethylammonio]-1-propanesulfonate, (*w*/*v*)). Protein concentration was determined using the 2D-Quant Kit (GE Healthcare; Piscataway, NJ, USA) using BSA as standard. This extraction method yielded 0.4–0.6 mg protein per biological replicate. Remaining processing steps and subsequent analysis protocols utilized LC-MS grade solvents (Spectrum Chemicals, New Brunswick, NJ, USA) and acids (Sigma-Aldrich, St. Louis, MO, USA), performed using equal protein (or peptide) amounts.

Disulfide bonds were reduced using 5 mM Tris(2-carboxyethyl)phosphine hydrochloride at room temperature for 30 min and then free sulfhydryls were alkylated using 50 mM iodoacetamide at room temperature in the dark for 40 min. Samples were diluted with 50 mM ammonium bicarbonate to a urea concentration of 1.6 M and trypsin (Promega, Madison, WI, USA) was added at a 50:1 (*w*/*w*) protein to trypsin ratio and incubated at 37 °C for 16 h. Reactions were quenched by the addition of TFA to a final concentration of 1% (*v*/*v*). Digestions were desalted utilizing Sep-Pak® Vac 1 cc/50 mg C_18_ cartridges (Waters Corporation, Milford, MA, USA) and a vacuum manifold. Digestions were brought to 0.2% (*v*/*v*) trifluoroacetic acid (TFA) and the pH was further adjusted to below 3 by the addition of formic acid if necessary. Cartridges were conditioned and equilibrated with methanol (1 mL), followed by water:acetonitrile (1 mL, 98:2 *v*/*v*, containing 0.1% TFA). 

Samples were evaporated to near dryness using a vacuum centrifuge and then resuspended in 1 mL 95% water, 5% acetonitrile (ACN), 0.1% TFA by sonication for 20 min. After centrifugation, the pH was adjusted to ≤3 if necessary using 5% TFA. C18RP-SPE was performed using Sep-Pak 1cc C18 cartridges (Waters Corporation). Cartridges were washed using 2 mL of MeOH and equilibrated with 4 mL 0.1% TFA. Samples were loaded using gravity flow and then the original tubes were rinsed with 1 mL 95% water, 5% ACN, 0.1% TFA, and the rinse was also loaded. Cartridges were then washed with 3 mL 95% water, 5% ACN, and 0.1% TFA. Peptides were eluted using 2 mL 60% ACN, 0.1% TFA.

ACN was removed using a vacuum centrifuge and samples were diluted to 4 mL using 10 mM KH_2_PO_4,_ pH 3.0, 25% MeOH. Strata-X-C 33u strong cation-exchange cartridges (SCX; Phenomenex, Torrance, CA, USA) were washed using 1 mL MeOH and then equilibrated with 3 mL 10 mM KH_2_PO_4,_ pH 3.0, 25% MeOH. After centrifugation of the samples, the pH was checked and adjusted to ≤3 using H_3_PO_4_ if necessary. Samples were loaded using gravity flow and then the original tubes were rinsed with 1 mL 10 mM KH_2_PO_4,_ pH 3.0, 25% MeOH and that rinse was added to the cartridge. Cartridges were washed with 3 mL 10 mM KH_2_PO_4,_ pH 3.0, 25% MeOH. Peptides were eluted in six steps using increasing concentrations of KCl in 10 mM KH_2_PO_4,_ pH 3.0, 25% MeOH: 25 mM, 50 mM, 75 mM, 150 mM, 350 mM, and 1 M. ACN was removed using a vacuum centrifuge and samples were desalted using C18-RP SPE as described above. ACN was removed using a vacuum centrifuge and samples were then freeze-dried and stored at −80 °C until further use. 

### 2.3. LC-MS Analysis

Each SCX fraction was analyzed using an Acquity I-class UPLC interfaced with a Synapt G2-S mass spectrometer (Waters Corporation) employing a randomized sample queue. The mobile phases were Solvent A (0.1% (*v*/*v*) formic acid in LC/MS grade water) and solvent B (0.1% (*v*/*v*) formic acid in LC/MS grade acetonitrile (Spectrum Chemicals)). The separation was performed using a CSH130 C18 1.7 µm, 1.0 × 150 mm column (Waters Corporation) at 50 µL/min using a 110-minute gradient from 3–40% solvent B. The column temperature was maintained at 45 °C.

Column effluent was analyzed by mass spectrometry using the HDMS^E^ (high-definition mass spectrometry) with alternating scans utilizing low and elevated collision energies acquisition method in continuum positive ion “resolution” MS mode. Source conditions were as follows: capillary voltage, 3.0 kV; source temperature, 120 °C; sampling cone, 60 V; desolvation temperature, 350 °C; cone gas flow, 50 L/h; desolvation gas flow, 500 L/h; nebulizer gas flow, 6 bar. Both low energy (4 V and 2 V in the trap and transfer region, respectively) and elevated energy (4 V in the trap and ramped from 20 to 50 V in the transfer region) scans were 1.2 sec each for the *m*/*z* range of 50 to 1800. For ion mobility separation, the IMS and transfer wave velocities were 600 and 1200 m/sec, respectively. Wave height within the ion mobility cell was ramped from 10 to 40 V. For lock-mass correction, a 1.2 second low energy scan was acquired every 30 sec of a 100 fmol/µL [Glu1]-fibrinopeptide B (Waters Corporation) solution (50:50 ACN:H_2_O supplemented with 0.1% formic acid) infused at 10 µL/min introduced into the mass spectrometer through a different source which was also maintained at a capillary voltage of 3.0 kV. The data for lock-mass correction was collected and applied during data processing.

### 2.4. Data Analysis

Mass spectrometric data were processed and analyzed utilizing ProteinLynx Global Server v. 3.0.2 (PLGS, Waters Corporation). Chromatographic and mass spectrometric peak width resolutions were automatically determined using the software. Mass values were lock-mass corrected based on the exact *m*/*z* value of the +2 charge state of [Glu1]-fibrinopeptide B (785.842). Peaks were defined based on the low energy, elevated energy and bin intensity thresholds of 300, 20, and 1500 counts, respectively. Peak lists generated for each of the six SCX fractions from a sample were merged using PLGS after processing. The final peak list for each sample was then searched against a protein database containing the complete *A. thaliana* proteome including isoforms downloaded from UniProt (www.uniprot.org) and a randomized decoy entry for each real entry appended using PLGS. Workflow parameters for the protein identification searches were two possible missed cleavages utilizing trypsin as the protease, a fixed modification of carbamidomethylation of cysteine, possible modifications of carbamylation of lysine and the N-terminus of peptides, and oxidation of methionine. The software automatically determined peptide and peptide fragment mass tolerances and protein identifications were limited to less than 5% FDR. Information from the PLGS analysis was then processed and summarized using IsoQuant [12] to resolve homology and quantitate proteins. Analysis in IsoQuant limited both peptide and protein identifications to less than 1% FDR. Protein quantitation using the Top3 method was limited to unique and razor peptides excluding those that resulted from in-source decay and allowed different peptides to be utilized for protein quantitation across samples. The mass spectrometry proteomics data have been deposited to the ProteomeXchange Consortium via the PRIDE [15] partner repository with the dataset identifier PXD001400.

## 3. Results and Discussion

### 3.1. Proteomic Overview

To compare the auxin response of the plant root proteome relative to that of the transcriptome, *Arabidopsis* Col-0 seedlings were grown in continuous light on agar plates for 5–6 d following germination and then transferred to agar plates plus or minus 1 µM IAA for 8, 12, or 24 h in biological triplicate, identical to the procedure described for two recent transcriptomic analyses [13,14]. Under these conditions the auxin-treated seedlings exhibited the temporal inhibition of primary root elongation and increases in lateral root initiation as reported previously [13], where elongation was significantly different from controls at 30 min and lateral root initiation was statistically significant by 8 h. Roots were excised into liquid nitrogen with protein lysates prepared by a modified phenol-methanol method. The lysates were then reduced, alkylated, and digested with trypsin to generate the peptides used for mass spectrometry-based proteomic analysis.

The mass spectrometer used in this study, a Synapt G2-S (Waters, Manchester, UK), can perform DIA analyses in two modes, so-called MS^E^ and HDMS^E^. In MS^E^ mode, the instrument cycles between low- and high-energy scans, which first record intact precursor masses and then apply a ramp of collision energy to generate fragment ions [9]. The HDMS^E^ mode includes separation of ions using ion-mobility, which leads to increased accuracy of quantitation and a higher dynamic range for peptide detection [10,16,17]. To confirm that the ion mobility mode (HDMS^E^) is superior to MS^E^ in terms of protein identifications for protein lysates from *Arabidopsis* roots, we analyzed unfractionated lysates at a constant loading level of 1 μg and varied the chromatographic separation times and solvent flow rates. We observed that using the same separation gradient and flow rate, HDMS^E^ yielded at least 45% more protein identifications than MS^E^ for unfractionated protein lysates from *Arabidopsis* roots (Table S1). Hence, all work reported here was performed in HDMS^E^ mode.

To assess the technical variation of the LC-MS set up, unfractionated protein lysates were analyzed using duplicate injections of the same sample. There were 1445 proteins identified in the first technical replicate and 1442 in the second technical replicate, with 1285 proteins in common between the duplicate injections (Figure S1). Given this level of technical reproducibility, experiments in biological triplicate with random duplicate injections were used in combination with offline fractionation (strong-cation exchange chromatography, SCX), with the resulting six fractions analyzed using UPLC-HDMS^E^. Three timepoints in biological triplicate, plus and minus auxin, provided 18 samples for SCX fractionation, which resulted in 108 individual LC-MS samples analyzed in duplicate (216 LC-MS runs employing 2 h gradients).

**Figure 1:**
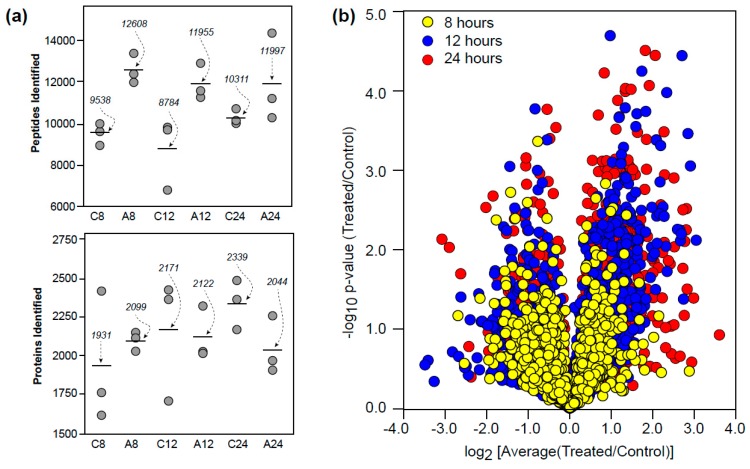
Proteomic overview. (**a**) Peptides and proteins identified in control (C8, C12, C24) and auxin-treated (A8, A12, A24) roots at 8, 12, and 24 h. (**b**) Proteins identified during the time course experiment in relation to ion intensity differences and *p*-value (statistical significance).

Approximately 2000 proteins were identified from over 10,000 peptides using the offline SCX and UPLC-HDMS^E^ protocol at a 1% protein FDR (Figure 1a). These identifications and associated ion intensities were then processed using the open-source ISOQuant program [12] to provide relative abundances based upon ion intensity. Differences in ion intensities for each time point and the significance of these changes across replicates is shown in Figure 1b. The data obtained is available via the PRIDE component of ProteomeXchange (PXD001400).

### 3.2. Temporal Differences

**Figure 2:**
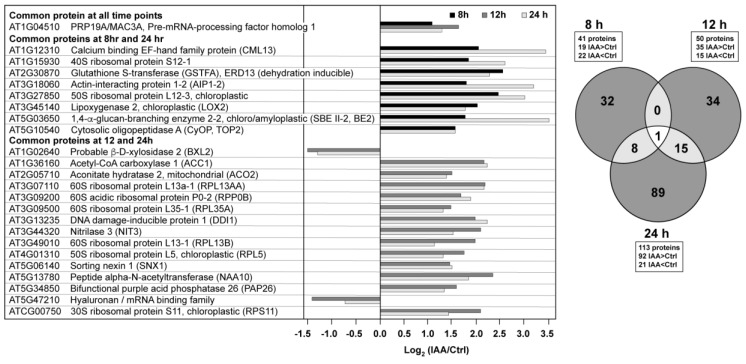
Auxin elicits time-dependent changes in the root proteome. The full list of proteins can be found in the Supplemental Materials (Table S3).

There were 41 proteins at the 8 h time point that met the criteria established for both fold change (treated/control <0.7 or >1.43) and significance (*p*-value < 0.05) (Figure 2). This increased to 50 proteins after 12 h and 113 proteins after 24 h. At the longer time points there was a shift toward higher protein abundances in the auxin-treated samples. There was little overlap between the proteins across the time series (Figure 2), with only one protein changing significantly across all three time points. This protein, PRP19/MAC3A (AT1G04510), is a putative E3 ubiquitin ligase with WD40 repeats associated with both innate immunity [18], the spliceosome [19], and cell wall synthesis [20,21]. 

There were 15 overlapping proteins when comparing the 12 and 24 h time points, with 13 of these being in higher abundance at both time points. Several of these auxin-induced proteins have already been implicated in lateral root processes. At5g13780 encodes a peptide N-terminal acetyltransferase (NAA10). Downregulation of NAA10 leads to decreased lateral root growth density and longer primary roots under non-stressed conditions [22], suggesting that IAA modulates protein turnover via the N-terminome [23]. Acetyl CoA carboxylase (ACC1, PASTICCINO3, PAS3), which uses an ATP-dependent carboxylation reaction to form malonyl CoA (the rate limiting step of lipid biosynthesis), was also increased in abundance at both 12 and 24 h. Roudier et al. reported that very long chain fatty acids (VLCFAs) are involved in polar auxin transport, as well as lateral root organogenesis [24]. Two other proteins are associated with auxin processing or transport, nitrilase (NRL3), which has been shown to catalyze the hydrolysis of indole-3-acetonitrile (IAN) to indole-3-acetic acid (IAA) in roots, with the exception of the root tip [25], and sorting nexin 1 (SNX1), which is known to be phosphorylated [6] and associated with the auxin response [26], potentially through modulating PIN1/2 protein levels at the plasma membrane [27,28].

Six ribosomal proteins were found in higher abundance upon auxin exposure at both the 12 and 24 h time points, with two annotated as chloroplastic. Ribosomal proteins are known translational regulators of the auxin response and provide downstream regulation of the lipid-based metabolic processes involved in tissue differentiation and trafficking [29]. In fact, a knockout mutation for the 60S ribosomal protein L4-2 gene, RPL4D, provides a root phenotype similar to that of exogenous auxin [29]. Although this protein was not among the six with altered abundance in the current study, of the 1800 genes regulated by an rpl4d knockout in seven-day-old *Arabidopsis* seedlings, 91 encoded proteins were identified and quantified in our investigation, with eight changing significantly in relative abundance (Table 1). While the time-scale, tissues, and growth conditions are different, all mRNAs were lower in abundance, whereas the proteins observed in our study were variable with respect to the direction of change.

The three other proteins that exhibited elevated levels at both 12 and 24 h were mitochondrial aconitate hydratase 2 (ACO2), DNA damage-inducible protein 1 (DDI1), and the purple acid phosphatase, PAP26. ACO2 is bifunctional protein that serves as a key enzyme in the TCA cycle, while also having a non-catalytic role in mediating the oxidative stress response [30]. The DDI1 protein is annotated as an aspartic-type endopeptidase that functions as a ubiquitin receptor, which suggests changes in proteasome activity in response to exposure to exogenous auxin [31]. PAP26, a glycoprotein associated with maintenance of intracellular phosphate levels, was also increased in abundance in the presence of auxin [32,33,34]. Previous work indicated that transcriptional controls exert little influence on PAP26 levels relative to those associated with translation and/or proteolysis [34]. 

The last two proteins common to the 12 and 24 h time points, AT5G47210 and BXL2 were lower in abundance in the presence of exogenous auxin. AT5G47210 is annotated as a hyaluronan/mRNA binding protein that was identified in the plasma membrane proteome [35]. BXL2 is presently annotated as an arabinofuranosidase/xylosidase that was originally identified from mature stems as a secreted glycosylated protein [36]. Partially redundant with BXL1, *bxl1* and *bxl2* double mutants have shortened siliques and curled leaf edges [37]; there is no published information with respect to its role in roots and/or the effects of auxin. 

Eight proteins were found in higher abundance in both the 8 and 24 h time points (Figure 2). The cytosolic oligopeptidase A (CyOP, TOP2) is in the zincin-like metalloprotease protein family, an enzyme inhibited by salicylic acid and upregulated by infection. The protein is thought to be involved in cytosolic peptide degradation processes [38]. The glutathione-S-transferase protein, GSTF10, is also known as ERD13 (EARLY RESPONSE TO DEHYDRATION 13) based upon its increase at the transcript level during drought stress, although its transcript also increases in the presence of auxin [39]. CML13 binds calcium but has no established role to date [40]. LOX2 performs the entry reaction to the jasmonic acid biosynthetic pathway and is a 13S-lipoxygenase that prefers linolenic acid as the substrate. Interestingly, the enzyme is localized to chloroplasts and can be induced by leaf wounding [41]. The other proteins were an actin binding protein (AIP1-2) known to be expressed in multiple cell types [42], and a starch branching enzyme (SBE II-2, BE2) whose translated product contains a signal peptide for chloroplast localization. The transcript is expressed throughout the plant and highest in seedlings and cauline leaves. Modifications in amylopectin structure were detected in a SBE2 mutant [43]. 

### 3.3. Small Secretory Peptides (SSPs) and Putative Cell Wall Synthesis-Related Proteins

The development of a lateral root requires extensive remodeling of both cells and the extracellular matrix (or apoplast), processes controlled to some degree by small secretory peptides (SSPs) [44]. Four SSPs were identified in our proteomic investigation (AT2G38540/LTP1, AT3G08770/LTP6, AT3G51600/LTP5, AT2G13820/XYP2), which are classified as protease inhibitors and lipid transfer proteins (LTPs) [44,45]. Three of the four proteins (LTP1, 5, and 6) were observed in all samples with LTP1 increasing significantly at the 12 and 24 h time points upon exposure to auxin. LTP1 was recently shown to function in the ethylene response pathway to regulate the ethylene receptor ETR1 [46]. A *ltp1* knockout is hypersensitive to 1-aminocyclo-propanecarboxylic acid (ACC) whereas an overexpressor is insensitive. LTP1 co-localizes with lipids within the cell wall, where changes in shape, volume, or polarity occur [47], all events associated with lateral root formation. 

In addition to the cell wall protein, PAP26, mentioned above, a recent search of protein-protein interaction databases for cell wall synthesis-related proteins provided a list of 42 candidates [20]. Five of these were identified at all time points and conditions (Table S4) with only MAC3A/PRP19 meeting the criteria for fold change and *p*-value across the experimental time window (Table S3). As stated earlier, this protein is presently annotated as a ubiquitin ligase with WD40 repeats, and is associated with both innate immunity [18], the spliceosome [19], and cell wall synthesis [20,21]. 

### 3.4. Proteins Associated with Mobile mRNAs

**Figure 3:**
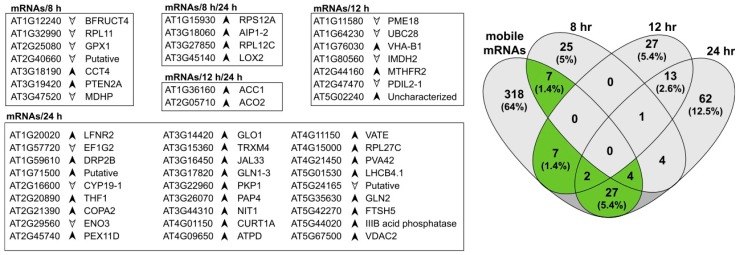
Proteins identified as significantly changing and also classified as being encoded by mobile mRNAs. The total number of proteins classified as mobile mRNAs increased with time and are predominantly plastid-related. Arrows indicate protein abundance in auxin-treated roots relative to controls.

Phloem-based mRNA transport between roots and aerial tissues has been measured in *Arabidopsis*, with over 2000 genes identified as encoding mobile mRNAs, predominantly moving from shoot to root [48]. We identified 365 proteins that are encoded by mobile mRNA genes in root tissues, with more than half of these proteins (52%) annotated in DAVID [49] as being associated with chloroplasts. From the list of 365 proteins, 47 were identified as significantly changing in relative levels upon exposure to exogenous auxin (Figure 3). There was little overlap across three time points with an increasing number of differences at the later time point. At 8 h there were eleven, six of which were higher in abundance, including the aforementioned chloroplastic lipoxygenase (LOX2), an enzyme that performs the entry reaction into the jasmonic acid pathway. At 12 h there were nine proteins potentially derived from mobile mRNAs, including a pectin methylesterase with known ribosome inhibition activity [50]. At 24 h there were 33 proteins, a much larger total number that may reflect the kinetics of mRNA transport and translation.

### 3.5. Future Directions

Auxin regulates many aspects of plant biology. Treatment with exogenous auxin ablates root elongation [51], induces lateral root formation, and globally affects the levels of transcripts [13,52,53,54]. Treatment with exogenous auxin stimulates a rapid increase in the GH3, SAUR, and AUX/IAA transcript families [55] and precipitates global transcriptional changes that peak from 2 to 8 h and return to baseline around 12 to 24 h [13]. These changes correlate at the physiological level with a rapid ablation of root elongation and a slower (around 8 h) induction of lateral root formation. In order to better understand the auxin response at the protein level, we exposed *Arabidopsis* seedlings to 1 μM IAA for 8, 12, or 24 h and analyzed root proteome profiles using UPLC-HDMS^E^. The results indicated that auxin signaling over a time course occurs through the regulation of proteins representing similar biological functions although there was minimal overlap between the proteins identified over the three time points.

Only one protein passed the established criteria for abundance change and significance at all three time points. PRP19/MAC3A was predicted as a cell wall protein and this work supports that claim. Proteins such as PRP19 contain WD40 repeats, structures that permit these proteins to act as molecular scaffolds for complex biochemical processes, such as forming and extending a lateral root [21]. Prp19 (E3 ubiquitin ligase Precursor RNA Processing 19) is also the namesake of NineTeen Complex (NTC), an assembly involved in formation of an active spliceosome, a process that involves protein arginine dimethylation via PRMT5 [19]. The role of PRP19 in lateral root formation and its relation to auxin-regulated processes is worthy of further investigation.

An enrichment of proteins involved in the response to light was observed by treatment with exogenous auxin, with 50 of the 92 proteins found in higher abundance at 24 h annotated in DAVID as a component of the chloroplast. Roots generally bend away from a light source (negative phototropism) due to a mechanism involving auxin, cytokinin, ROS, and flavonols [56]. The current light avoidance model posits that light induces localized flavonol production via a cytokinin-SHY2 pathway. An increase in the local flavonol level leads to decreased auxin transport as well as O_2_^−^ levels, which will favor cell elongation over proliferation. The root then bends away from the light source in the direction opposite to that of the high flavonol levels. Light avoidance was not possible in the system used in our work, conditions that lead to decreased root length and an increased number of lateral roots with auxin supplementation [13]. We also aimed to replicate the conditions used in a prior microarray study to determine whether changes in transcript levels were reflected at the protein level, and vice versa. Considering the complexity of the hormone-light axis in root development, further advances in our understanding of hormonal regulation of the root proteome may benefit from utilization of dark-grown root systems as recently reported by Silva-Navas and coworkers [56]. Such studies may remove the changes in chloroplast-related processes, revealing changes more directly related to belowground growth processes.

The plant root is a dynamic structure that is continuously modified in a temporal and spatial manner in order to respond to environmental stimuli, including auxin [57,58]. We observed that treatment with IAA for 8, 12, or 24 h affects proteins of similar biological processes, but generally not the same protein at more than one time point. In contrast to these results, analyses of changes in gene transcription in light-grown roots over a similar time-course reveals that auxin affects the abundance of many transcripts over a longer time period [13]. Of the 1276 genes identified as differentially expressed over a 24 h timeframe, only 108 of the proteins identified in this experiment overlapped with no significant enrichment of any biological processes when the list was queried against DAVID. When the proteome was evaluated using the clusters identified in the transcriptional analyses [13], again no significant enrichment was observed. Any observed proteome is dependent upon the methodology used for isolation and analysis, hence different strategies often yield complementary proteomes [59,60]. The correlative aspects of transcript and protein abundances continues to be a formidable challenge in plant systems biology [61,62]. That our study implicates several ribosomal and spliceosome proteins as being involved in auxin stimulation supports evaluating these core processes to better understand the fate of a transcript after formation and the downstream processes that modulate protein half-life. When correlated with specific cell types, post-translational modifications and metabolite levels, a more complete picture of root growth and development will begin to emerge.

## 4. Conclusions

The present study examined the effect of exogenous IAA on the *Arabidopsis* root proteome at three time points (8, 12, and 24 h). IAA affected the abundance of proteins involved in post-transcriptional events, light-related processes, and the maintenance and formation of cell walls. A number of these proteins were previously implicated auxin-mediated processes. Several proteins associated with mobile mRNAs were also identified. The correlation between protein and transcript levels under the same experimental regime was poor. 

## Figures and Tables

**Table 1:** Proteins implicated in the auxin response related to changes in ribosomal protein RPL4d levels. None of the proteins met the confidence threshold at the 12 h time point.

TAIR ID	LogFC ^1^	UniProt	Protein Names	8 h	24 h
AT1G06430	0.706	Q8W585	ATP-dependent Zn metalloprotease FTSH 8	0.442	n.s. ^2^
AT1G12240	0.767	Q39041	Acid β-fructofuranosidase 4, vacuolar	0.584	n.s.
AT3G45140	0.741	P38418	Lipoxygenase 2, chloroplastic	2.011	1.762
AT4G21650	0.694	Q8GUK4	Subtilisin-like protease SBT3.13, secreted	0.224	n.s.
AT4G28400	0.609	Q93YW5	Protein phosphatase 2C 58 (AtPP2C58)	n.s.	2.361
AT5G22880	0.618	Q9FFC0	Histone H2B.10 (HTB2)	n.s.	3.504
AT5G24165	0.537	Q8LDQ8	At5g24165 (uncharacterized protein)	n.s.	0.244
AT5G48030	0.533	Q8GWW8	Chaperone protein dnaJ GFA2, mitochondrial	1.703	n.s.

^1^ Transcript fold change for seven-day old seedlings, *rpl4d* knockout vs. control [29]. ^2^ Not significant.

## Supplementary Materials

The following are available at http://www.mdpi.com/2227-7382/5/3/16: Table S1. HDMS^E^ yielded more protein identifications than MS^E^ for unfractionated Arabidopsis root protein lysates; Figure S1. Technical replicates for the UPLC-HDMS^E^ protocol using unfractionated protein lysates; Table S2. Pearson correlation table for biological and technical replicates; Table S3. Protein abundances at 8, 12, and 24 h; Table S4. Proteins identified and matched to cell wall proteins based upon protein interactions.
