# Supplementary material for: Exogenous Auxin Elicits Changes in the Arabidopsis thaliana Root Proteome in a Time-Dependent Manner
Source: Proteomes. 2017 Jul 10;5(3):16. doi: 10.3390/proteomes5030016 (PMC5620533; doi:10.3390/proteomes5030016)
Supplement: Supplementary file 1 [file proteomes-05-00016-s001.pdf]

SUPPLEMENTAL MATERIALS

Exogenous auxin elicits changes in the *Arabidopsis thaliana* root proteome in a time-dependent manner

William O. Slade[1], W. Keith Ray[2], Sherry B. Hildreth[1], Brenda S. J. Winkel[1], Richard F. Helm[2§]

[1]Department of Biological Sciences, Virginia Tech, Blacksburg, VA, USA
[2]Department of Biochemistry, Virginia Tech, Blacksburg, VA, USA

[§]Corresponding author (helmrf@vt.edu)

TABLE OF CONTENTS

Item	Description	Page
Table S1	HDMS^E yielded more protein identifications than MS^E for unfractionated Arabidopsis root protein lysates.	2
Figure S1	Technical replicates for the UPLC-HDMSE protocol using unfractionated protein lysates.	2
Table S2	Pearson correlation table for biological and technical replicates.	2
Table S3	Protein abundances at 8, 12, and 24 hours (A=auxin-treated, C=control).	3
Table S4	Proteins Identified and Matched to Cell Wall Proteins Based Upon Protein Interactions. Includes STRING-based protein interactions.	7

PRIDE Identifier: PXD001400 Effects of exogenous auxin on the *Arabidopsis thaliana* root proteome at 8, 12, and 24 hours post-treatment.

Table S1. Comparision of protein identifications between MS^E and $HDMS^E$ using PLGS 3.0 under constant loading level, differing gradient lengths, and differing flow rates.

Loading	Gradient length (min)	Flow rate (µL/min)	MSE Protein IDs	HDMSE Protein IDs
1 µg	100	50	393	866
1 µg	160	50	400	872
1 µg	200	50	254	792
1 µg	100	20	278	632
1 µg	160	20	345	624
1 µg	200	20	116	785

Figure S1. Comparison of the reproducibility of unfractionated technical replicates at the level of protein identifications using DIA-MS ($HDMS^E$ mode).

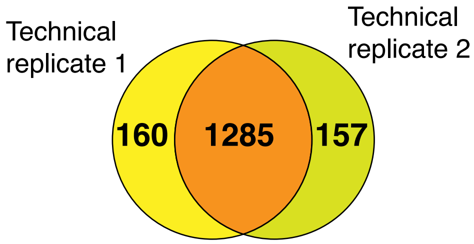

		Controls									Auxin Treated								
		8 hrs			12 hrs			24 hrs			8 hrs			12 hrs			24 hrs		
		1	2	3	1	2	3	1	2	3	1	2	3	1	2	3	1	2	3
Controls	C8 1	1.0000	0.8638	0.8822	0.8728	0.8538	0.8342	0.9042	0.8650	0.9096	0.8960	0.8923	0.8833	0.8177	0.8540	0.8830	0.8173	0.9039	0.9000
	C8 2	0.8638	1.0000	0.9061	0.8226	0.8299	0.8677	0.8742	0.8344	0.8034	0.8670	0.8778	0.9177	0.8989	0.8124	0.8532	0.9052	0.8997	0.8652
	C8 3	0.8822	0.9061	1.0000	0.8919	0.8264	0.8793	0.9111	0.8952	0.8710	0.9471	0.9506	0.9680	0.9278	0.9497	0.9429	0.9339	0.9582	0.9426
	C12 1	0.8728	0.8226	0.8919	1.0000	0.9037	0.9144	0.9482	0.9529	0.9101	0.8842	0.8734	0.8616	0.8446	0.8772	0.8352	0.8766	0.8681	0.8795
	C12 2	0.8538	0.8299	0.8264	0.9037	1.0000	0.9092	0.9227	0.9118	0.9356	0.8741	0.8302	0.8273	0.7898	0.8334	0.7643	0.8261	0.8414	0.8662
	C12 3	0.8342	0.8677	0.8793	0.9144	0.9092	1.0000	0.9243	0.9101	0.9079	0.8670	0.8746	0.8660	0.8497	0.8580	0.8024	0.8820	0.8708	0.8743
	C24 1	0.9042	0.8742	0.9111	0.9482	0.9227	0.9243	1.0000	0.9454	0.9288	0.9201	0.8953	0.8987	0.8403	0.8837	0.8700	0.8690	0.8975	0.9097
	C24 2	0.8650	0.8344	0.8952	0.9529	0.9118	0.9101	0.9454	1.0000	0.9129	0.9190	0.8742	0.8711	0.8465	0.8890	0.8301	0.8702	0.8652	0.9165
	C24 3	0.9096	0.8034	0.8710	0.9101	0.9356	0.9079	0.9288	0.9129	1.0000	0.9069	0.9075	0.8744	0.7972	0.8752	0.8304	0.8269	0.8848	0.9204
Auxin Treated	A8 1	0.8960	0.8670	0.9471	0.8842	0.8741	0.8670	0.9201	0.9190	0.9069	1.0000	0.9428	0.9428	0.8916	0.9295	0.9088	0.8928	0.9420	0.9629
	A8 2	0.8923	0.8778	0.9506	0.8734	0.8302	0.8746	0.8953	0.8742	0.9075	0.9428	1.0000	0.9571	0.8684	0.9349	0.9075	0.9011	0.9491	0.9486
	A8 3	0.8833	0.9177	0.9680	0.8616	0.8273	0.8660	0.8987	0.8711	0.8744	0.9428	0.9571	1.0000	0.9073	0.9315	0.9294	0.9248	0.9585	0.9494
	A12 1	0.8177	0.8989	0.9278	0.8446	0.7898	0.8497	0.8403	0.8465	0.7972	0.8916	0.8684	0.9073	1.0000	0.9247	0.8882	0.9614	0.9054	0.8723
	A12 2	0.8540	0.8124	0.9497	0.8772	0.8334	0.8580	0.8837	0.8890	0.8752	0.9295	0.9349	0.9315	0.9247	1.0000	0.9117	0.9158	0.9187	0.9278
	A12 3	0.8830	0.8532	0.9429	0.8352	0.7643	0.8024	0.8700	0.8301	0.8304	0.9088	0.9075	0.9294	0.8882	0.9117	1.0000	0.8873	0.9401	0.9170
	A24 1	0.8173	0.9052	0.9339	0.8766	0.8261	0.8820	0.8690	0.8702	0.8269	0.8928	0.9011	0.9248	0.9614	0.9158	0.8873	1.0000	0.9269	0.8845
	A24 2	0.9039	0.8997	0.9582	0.8681	0.8414	0.8708	0.8975	0.8652	0.8848	0.9420	0.9491	0.9585	0.9054	0.9187	0.9401	0.9269	1.0000	0.9553
	A24 3	0.9000	0.8652	0.9426	0.8795	0.8662	0.8743	0.9097	0.9165	0.9204	0.9629	0.9486	0.9494	0.8723	0.9278	0.9170	0.8845	0.9553	1.0000

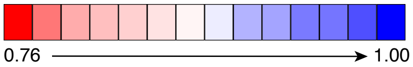

Averaged Pearson Correlations	
C8	0.8840
C12	0.9091
C24	0.9290
A8	0.9476
A12	0.9082
A24	0.9222
C8-A8	0.9111
C12-A12	0.8283
C24-A24	0.8845

Table S2. Pearson Coefficients Across Technical and Biological Replicates. The first column and row indicate biological replicates for combined LC-MS runs. Averaged Pearson Correlations between replicates are shown to the right. Calculated using the "CORREL" function in Excel.

Table S3. Protein abundances at 8, 12 and 24 hours (A=auxin-treated, C=control).

Protein Description (8 hours)	IEP	mw	max score	accession	reported peptides	sequence coverage	FDR level	UniProt ID	AVE C8	AVE A8	A8/C8 Avg	8hr p-value	UniProt Entry Name	Protein names (UniProt)
KH domain-containing protein	5.15	64664	405	AT2G25970.1	17	17.25	0.0118	O82762	758.0	2385.2	3.1466	0.0274	O82762_ARATH	F17H15.1/F17H15.1 (KH domain-containing protein) (Putative uncharacterized protein At2g25970)
ATGSTF10, ERD13, ATGSTF4, GSTF10 glutathione S-transferase PHI 10	5.35	24230	4115	AT2G30870.1	11	27.91	0	P42761	206.6	526.5	2.5489	0.0039	GSTFA_ARATH	Glutathione S-transferase F10 (AtGSTF10) (EC 2.5.1.18) (AtGSTF4) (GST class-phi member 10) (Protein EARLY RESPONSE TO DEHYDRATION 13)
RPL12-C ribosomal protein L12-C	5.37	19682	6895	AT3G27850.1	16	42.78	0	P36212	187.7	460.4	2.4526	0.0119	RK123_ARATH	50S ribosomal protein L12-3, chloroplastic (CL12-C)
MAC3A MOS4-associated complex 3A	6.16	57265	242	AT1G04510.1	6	10.71	0.0253	Q94BR4	435.5	914.8	2.1003	0.0287	PR19A_ARATH	Pre-mRNA-processing factor 19 homolog 1 (EC 6.3.2.-) (MOS4-associated complex protein 3A) (MAC protein 3A) (Plant U-box protein 59) (U-box domain-containing protein 59)
Calcium-binding EF-hand family protein	4.58	16517	7647	AT1G12310.1	9	33.11	0	Q94AZ4	191.6	390.0	2.0352	0.0031	CML13_ARATH	Probable calcium-binding protein CML13 (Calmodulin-like protein 13)
LOX2, ATLOX2 lipoxygenase 2	5.29	102616	616	AT3G45140.1	27	22.32	0.0037	P38418	544.0	1094.2	2.0113	0.0176	LOX2_ARATH	Lipoxygenase 2, chloroplastic (AtLOX2) (EC 1.13.11.12)
ATGSTF9, GLUTTR, ATGSTF7, GSTF9 glutathione S-transferase PHI 9	6.07	18679	1571	AT2G30860.2	9	34.34	0.0019	O80852	182.3	357.4	1.9604	0.0035	GSTF9_ARATH	Glutathione S-transferase F9 (AtGSTF9) (EC 2.5.1.18) (AtGSTF7) (GST class-phi member 9)
Ribosomal protein L7Ae/L30e/S12e/Gadd45 family protein	5.24	15719	4433	AT1G15930.1	6	40.28	0	Q9S9P1	86.7	160.1	1.8457	0.0218	RS121_ARATH	40S ribosomal protein S12-1
transducin family protein / WD-40 repeat family protein	5.52	66840	373	AT3G18060.1	17	24.96	0.0133	Q9LV35	438.3	789.2	1.8005	0.0016	AIP12_ARATH	Actin-interacting protein 1-2
SBE2.2 starch branching enzyme 2.2	5.45	92991	1842	AT5G03650.1	15	21.99	0.0019	Q9LZS3	698.6	1234.5	1.7672	0.0415	GLGB2_ARATH	1,4-alpha-glucan-branching enzyme 2-2, chloroplastic/amyloplastic (AtSBE II-2) (EC 2.4.1.18) (Branching enzyme 2) (AtBE2) (Starch-branching enzyme 2-2)
GFA2 gametophytic factor 2	9.08	50065	263	AT5G48030.1	9	23.9	0.0234	Q8GWW8	103.6	176.5	1.7033	0.0127	GFA2_ARATH	Chaperone protein dnaJ GFA2, mitochondrial (Chaperone protein dnaJ A30) (AtDjA30) (Gametophytic factor 2)
ATPE ATP synthase epsilon chain c	5.76	14556	4733	ATCG00470.1	8	46.21	0	P09468	36.7	61.4	1.6728	0.0341	ATPE_ARATH	ATP synthase epsilon chain, chloroplastic (ATP synthase F1 sector epsilon subunit) (F-ATPase epsilon subunit)
ATPEN2, PEN2 PTEN 2	5.16	67001	271	AT3G19420.1	10	29.62	0.0223	Q9LT75	84.1	130.5	1.5512	0.0430	PTN2A_ARATH	Phosphatidylinositol 3,4,5-trisphosphate 3-phosphatase and protein-tyrosine-phosphatase PTEN2A (EC 3.1.3.48) (EC 3.1.3.67) (Protein PHOSPHATASE AND TENSIN HOMOLOG 2-a) (AtPTEN2) (AtPTEN2a)
Zincin-like metalloproteases family protein	5.31	79272	1305	AT5G10540.1	10	10.56	0.003	Q949P2	302.6	469.3	1.5507	0.0122	COPDA_ARATH	Probable cytosolic oligopeptidase A (EC 3.4.24.70) (Thimet metalloendopeptidase 2) (Zincin-like metalloproteases family protein 2)
TCP-1/cpn60 chaperonin family protein	7.57	58232	301	AT3G18190.1	11	17.91	0.0176	Q9LV21	326.0	499.8	1.5331	0.0024	TCPD_ARATH	T-complex protein 1 subunit delta (TCP-1-delta) (CCT-delta) (Chaperonin CCT4)
NAI2 DNA topoisomerase-related	4.42	85245	1512	AT3G15950.1	43	51.81	0.0019	Q9LSB4	1187.9	1795.1	1.5111	0.0108	NAI2_ARATH	TSA1-like protein (Protein NAI2)
ATP binding;leucine-tRNA ligases;aminoacyl-tRNA ligases;nucleotide binding;ATP binding;aminoacyl-tRNA ligases	5.64	124592	694	AT1G09620.1	27	20.44	0.0036	F4I116	1480.5	2235.6	1.5101	0.0288	SYLC_ARATH	Leucine--tRNA ligase, cytoplasmic (EC 6.1.1.4) (Leucyl-tRNA synthetase) (LeuRS)
CHLI1, CH42, CH-42, CHL11, CHLI-1 P-loop containing nucleoside triphosphate hydrolases superfamily protein	6.04	46555	1970	AT4G18480.1	15	17.92	0.0019	P16127	450.7	679.0	1.5066	0.0152	CHLI1_ARATH	Magnesium-chelatase subunit ChlI-1, chloroplastic (Mg-chelatase subunit I-1) (EC 6.6.1.1) (Mg-protoporphyrin IX chelatase subunit ChlI-1) (Protein CHLORINA 42)
MGP1 copper ion binding;cobalt ion binding;zinc ion binding	9.5	25160	2068	AT2G21870.2	16	39.09	0	Q9SJ12	127.4	190.3	1.4936	0.0337	ATP7_ARATH	Probable ATP synthase 24 kDa subunit, mitochondrial
VCS Transducin/WD40 repeat-like superfamily protein	5.64	143004	806	AT3G13300.2	30	23.15	0.0036	Q9LTT8	798.4	549.4	0.6881	0.0376	VCS_ARATH	Enhancer of mRNA-decapping protein 4 (Protein VARICOSE)
MDH malate dehydrogenase	8.76	42634	3541	AT3G47520.1	25	47.89	0	Q9SN86	735.7	501.5	0.6816	0.0043	MDHP_ARATH	Malate dehydrogenase, chloroplastic (EC 1.1.1.37) (pNAD-MDH)
ARA2, ATRABA1A, ATRAB11E, ARA-2 Ras-related small GTP-binding family protein	6.52	24098	455	AT1G06400.1	3	14.35	0.0111	P28185	41.5	27.6	0.6663	0.0486	RAA1A_ARATH	Ras-related protein RABA1a (AtRABA1a) (Ras-related protein Ara-2) (Ras-related protein Rab11E) (AtRab11E)
TCP-1/cpn60 chaperonin family protein	5.2	51660	2533	AT1G24510.2	17	26.36	0	O04450	836.7	548.9	0.6560	0.0332	TCPE_ARATH	T-complex protein 1 subunit epsilon (TCP-1-epsilon) (CCT-epsilon) (Chaperonin CCT5)
A37, ATPDX1.2, PDX1.2 pyridoxine biosynthesis 1.2	5.32	34064	627	AT3G16050.1	6	19.11	0.0037	Q9ZNR6	82.4	54.0	0.6552	0.0473	PDX12_ARATH	Pyridoxal 5'-phosphate synthase-like subunit PDX1.2 (AtPDX1.2) (AtPDX1;3)
Lactate/malate dehydrogenase family protein	5.68	37327	4883	AT5G56720.1	3	8.85	0	Q9FJU0	272.1	172.6	0.6344	0.0095	Q9FJU0_ARATH	Malate dehydrogenase (EC 1.1.1.37)
PRPL11 plastid ribosomal protein l11	10.13	23319	4738	AT1G32990.1	18	71.17	0	Q9MAP3	848.0	523.9	0.6178	0.0493	RK11_ARATH	50S ribosomal protein L11, chloroplastic (CL11)
CAC2 acetyl Co-enzyme a carboxylase biotin carboxylase subunit	6.9	58901	2539	AT5G35360.1	25	46.93	0	O04983	819.6	484.5	0.5911	0.0404	ACCC_ARATH	Biotin carboxylase, chloroplastic (EC 6.3.4.14) (Acetyl-CoA carboxylase subunit A) (ACC) (EC 6.4.1.2)
ATBETAFRUCT4, VAC-INV Glycosyl hydrolases family 32 protein	5.25	74129	311	AT1G12240.1	12	23.8	0.0176	Q39041	595.4	347.8	0.5842	0.0005	INVA4_ARATH	Acid beta-fructofuranosidase 4, vacuolar (At beta fruct4) (AtBETAFRUCT4) (EC 3.2.1.26) (Acid invertase 4) (AI 4) (Acid sucrose hydrolase 4) (Vacuolar invertase 4) (Inv-V4) (VAC-INV 4) (VI 4)
HSP91 heat shock protein 91	4.96	92549	857	AT1G79930.1	6	8.9	0.0036	Q9S7C0	245.9	140.7	0.5721	0.0423	HSP7O_ARATH	Heat shock 70 kDa protein 14 (Heat shock protein 70-14) (AtHsp70-14) (Heat shock protein 91)
AHA3, ATAHA3, HA3 H(+)-ATPase 3	6.75	104963	262	AT5G57350.1	4	5.27	0.0234	P20431	1080.3	588.6	0.5448	0.0410	PMA3_ARATH	ATPase 3, plasma membrane-type (EC 3.6.3.6) (Proton pump 3)
Ribosomal protein L30/L7 family protein	10.34	28228	4369	AT2G01250.1	5	17.77	0	P60040	369.9	188.9	0.5106	0.0097	RL72_ARATH	60S ribosomal protein L7-2
YebC-related	6.17	37042	330	AT2G25830.1	3	9.67	0.0167	O82314	130.9	64.4	0.4924	0.0216	U082_ARATH	Probable transcriptional regulatory protein At2g25830
APS2, ASA1 Pseudouridine synthase/archaeosine transglycosylase-like family protein	6.14	53752	235	AT1G19920.1	7	17.86	0.0261	Q43870	237.3	116.8	0.4922	0.0027	APS2_ARATH	ATP sulfurylase 2 (EC 2.7.7.4)
Nucleic acid-binding, OB-fold-like protein	7.22	42316	511	AT2G40660.1	14	34.7	0.0067	Q93VB0	167.8	77.8	0.4634	0.0401	Q93VB0_ARATH	Nucleic acid-binding, OB-fold-like protein (Putative methionyl-tRNA synthetase)
ATRABA4B, ATRAB11G, ATGB3, RABA4B RAB GTPase homolog A4B	5.73	24521	703	AT4G39990.1	5	23.66	0.0036	Q9SMQ6	55.7	25.5	0.4577	0.0287	RAA4B_ARATH	Ras-related protein RABA4b (AtRABA4b) (Ras-related protein GB3) (AtGB3) (Ras-related protein Rab11G) (AtRab11G)
FTSH8 FTSH protease 8	5.59	73370	2160	AT1G06430.1	9	14.01	0	Q8W585	840.3	371.4	0.4420	0.0197	FTSH8_ARATH	ATP-dependent zinc metalloprotease FTSH 8, chloroplastic (AtFTSH8) (EC 3.4.24.-)
ATGPX1, GPX1 glutathione peroxidase 1	9.76	26187	953	AT2G25080.1	9	27.12	0.0036	P52032	303.1	116.2	0.3833	0.0171	GPX1_ARATH	Phospholipid hydroperoxide glutathione peroxidase 1, chloroplastic (PHGPx) (EC 1.11.1.12)
Co-chaperone GrpE family protein	9.86	31337	1097	AT1G36390.1	3	11.11	0.003	Q9C8X4	44.1	16.4	0.3724	0.0020	Q9C8X4_ARATH	GrpE protein homolog
RmlC-like cupins superfamily protein	5.76	38539	333	AT1G07750.1	3	11.52	0.0167	Q9LQQ3	64.2	21.6	0.3371	0.0194	Q9LQQ3_ARATH	At1g07750/F24B9_13 (Cupin domain-containing protein) (F24B9.13 protein) (Putative globulin protein)
NUP50 (Nucleoporin 50 kDa) protein	4.62	46820	189	AT1G52380.1	3	8.41	0.0465	Q9C829	704.4	200.0	0.2840	0.0498	NU50A_ARATH	Nuclear pore complex protein NUP50A (Nucleoporin 50A)
Subtilase family protein	7.04	81993	275	AT4G21650.1	5	8.49	0.0206	Q8GUK4	1000.8	224.1	0.2239	0.0428	SBT3D_ARATH	Subtilisin-like protease SBT3.13 (EC 3.4.21.-) (Subtilase subfamily 3 member 13) (AtSBT3.13)

Protein Description (12 hours)	IEP	mw	max score	accession	reported peptides	sequence coverage	FDR level	UniProt	AVE C12	AVE A12	A12/C12 Avg	12 p-value	UniProt Entry Name	Protein names (UniProt)
Major facilitator superfamily protein	7.50	56632	180	AT5G26250.1	2	6.51	0.0474	Q9SBA7	324.8	2094.2	6.4471	0.0064	STP8_ARATH	Sugar transport protein 8 (Hexose transporter 8)
Zinc-binding ribosomal protein family protein	10.72	10673	1953	AT3G10950.1	4	17.39	0.0019	Q9SRK6	27.5	174.7	6.3469	0.0078	R37A1_ARATH	Putative 60S ribosomal protein L37a-1
Putative 60S ribosomal protein L37a-1	5.75	31530	817	AT3G55250.1	3	13.36	0.0036	Q9M3C6	81.8	515.2	6.2998	0.0057	Q9M3C6_ARATH	Putative calcium homeostasis regulator (Putative uncharacterized protein At3g55250) (Putative uncharacterized protein T26I12.130) (Uncharacterized protein)
Acyl-CoA N-acyltransferases (NAT) superfamily protein	7.01	22057	1996	AT5G13780.1	8	53.13	0	Q9FKI4	210.9	1064.3	5.0463	0.0001	Q9FKI4_ARATH	Peptide alpha-N-acetyltransferase (Putative uncharacterized protein MXE10.5) (Silencing group B protein) (Similarity to N-acetyltransferase)
ATGSR1, GLN1;1, GSR 1, ATGLN1;1 glutamine synthase clone R1	5.13	39343	1938	AT5G37600.1	2	3.65	0.0019	Q56WN1	39.7	197.7	4.9779	0.0091	GLN11_ARATH	Glutamine synthetase cytosolic isozyme 1-1 (EC 6.3.1.2) (Glutamate--ammonia ligase GLN1;1) (GLN1;1)
Dihydrolipoamide succinyltransferase	7.69	39703	285	AT4G26910.3	3	8.22	0.0196	Q8H107	17.1	82.7	4.8329	0.0038	ODO2B_ARATH	Dihydrolipoyllysine-residue succinyltransferase component of 2-oxoglutarate dehydrogenase complex 2, mitochondrial (EC 2.3.1.61) (2-oxoglutarate dehydrogenase complex component E2-2) (OGDC-E2-2) (Dihydrolipoamide succinyltransferase component of 2-oxoglutarate dehydrogenase complex 2) (E2K-2)
NDPK2, NDPK1A, NDPK IA IA, NDPK IA, ATNDPK2 nucleoside diphosphate kinase 2	9.27	25836	3037	AT5G63310.1	13	42.86	0	O64903	245.6	1163.5	4.7372	0.0029	NDK2_ARATH	Nucleoside diphosphate kinase II, chloroplastic (NDK II) (NDP kinase II) (NDPK II) (NDPK Ia) (EC 2.7.4.6)
STV1, RPL24B, RPL24 Ribosomal protein L24e family protein	11.23	18746	1006	AT3G53020.1	6	17.18	0.0036	P38666	38.3	174.8	4.5626	0.0005	RL242_ARATH	60S ribosomal protein L24-2 (Protein SHORT VALVE 1)
Ribosomal protein L13 family protein	10.83	23694	547	AT3G07110.1	7	20.87	0.0053	Q9SFU1	52.3	236.1	4.5132	0.0014	R13A1_ARATH	60S ribosomal protein L13a-1
ACC1, AT-ACC1, EMB22, GK, PAS3 acetyl-CoA carboxylase 1	6.07	252979	358	AT1G36160.1	31	16.1	0.0145	Q38970	1829.1	8193.9	4.4797	0.0035	ACC1_ARATH	Acetyl-CoA carboxylase 1 (AtACC1) (EC 6.4.1.2) (Protein EMBRYO DEFECTIVE 22) (Protein GURKE) (Protein PASTICCINO 3) [Includes: Biotin carboxylase (EC 6.3.4.14)]
PSBH photosystem II reaction center protein H	7.08	7702	1307	ATCG00710.1	4	28.77	0.003	P56780	27.8	118.6	4.2675	0.0038	PSBH_ARATH	Photosystem II reaction center protein H (PSII-H) (Photosystem II 10 kDa phosphoprotein)
RPS11 ribosomal protein S11	12.65	15138	4477	ATCG00750.1	5	17.39	0	P56802	39.3	167.0	4.2455	0.0004	RR11_ARATH	30S ribosomal protein S11, chloroplastic
NIT3, AtNIT3 nitrilase 3	5.55	38364	1516	AT3G44320.1	8	13.58	0.0019	P46010	68.5	290.2	4.2336	0.0004	NRL3_ARATH	Nitrilase 3 (EC 3.5.5.1)
ubiquitin family protein	4.63	45500	210	AT3G13235.3	5	9.93	0.034	F4JC86	134.0	527.9	3.9393	0.0029	F4JC86_ARATH	DNA damage-inducible protein 1

ATBBC1, BBC1, RSU2 breast basic conserved 1	11.46	23767	4950	AT3G49010.1	7	22.33	0	P41127	83.7	328.1	3.9212	0.0093	RL131_ARATH	60S ribosomal protein L13-1 (Protein BBC1 homolog)
NAD(P)-binding Rossmann-fold superfamily protein	6.20	27160	800	AT5G02240.1	11	37.15	0.0036	Q94EG6	59.5	211.4	3.5557	0.0002	Y5224_ARATH	Uncharacterized protein At5g02240
ATNADP-ME2, NADP-ME2 NADP-malic enzyme 2	5.97	64812	354	AT5G11670.1	9	13.78	0.0158	Q9LYG3	156.5	554.5	3.5429	0.0060	MAOP2_ARATH	NADP-dependent malic enzyme 2 (AtNADP-ME2) (NADP-malic enzyme 2) (EC 1.1.1.40)
Ribosomal L5P family protein	10.43	28458	579	AT4G01310.1	20	59.54	0.0045	O04603	77.7	258.9	3.3301	0.0001	RK5_ARATH	50S ribosomal protein L5, chloroplastic
eIFiso4G2 MIF4G domain-containing protein / MA3 domain-containing protein	7.51	83460	680	AT2G24050.1	6	10.44	0.0036	O82233	380.4	1235.6	3.2482	0.0056	IF4G2_ARATH	Eukaryotic translation initiation factor isoform 4G-2 (eIF(iso)4G-2)
ATPase, V1 complex, subunit B protein	4.80	54222	4876	AT1G76030.1	6	10.29	0	P11574	174.7	567.3	3.2479	0.0058	VATB1_ARATH	V-type proton ATPase subunit B1 (V-ATPase subunit B1) (V-ATPase 57 kDa subunit) (Vacuolar H(+)-ATPase subunit B isoform 1) (Vacuolar proton pump subunit B1)
Ribosomal protein L10 family protein	4.57	30675	9150	AT3G09200.2	12	27.18	0	Q42112	134.6	426.7	3.1707	0.0074	RLA02_ARATH	60S acidic ribosomal protein P0-2
MAC3A MOS4-associated complex 3A	6.16	57265	242	AT1G04510.1	6	10.71	0.0253	Q94BR4	320.6	994.8	3.1032	0.0075	PR19A_ARATH	Pre-mRNA-processing factor 19 homolog 1 (EC 6.3.2.-) (MOS4-associated complex protein 3A) (MAC protein 3A) (Plant U-box protein 59) (U-box domain-containing protein 59)
PSBA photosystem II reaction center protein A	4.97	39051	3351	ATCG00020.1	7	13.31	0	P83755	368.3	1142.7	3.1029	0.0049	PSBA_ARATH	Photosystem II protein D1 (PSII D1 protein) (EC 1.10.3.9) (Photosystem II Q(B) protein)
MTHFR2 methylenetetrahydrofolate reductase 2	5.17	67430	1651	AT2G44160.1	18	33	0.0019	O80585	370.6	1136.4	3.0665	0.0002	MTHR2_ARATH	Methylenetetrahydrofolate reductase 2 (AtMTHFR2) (EC 1.5.1.20)
ATPAP26, PAP26 purple acid phosphatase 26	6.87	55067	391	AT5G34850.1	12	23.58	0.0118	Q949Y3	240.1	723.5	3.0132	0.0003	PPA26_ARATH	Bifunctional purple acid phosphatase 26 [Includes: Acid phosphatase (EC 3.1.3.2); Peroxidase (EC 1.11.1.7)]
tolB protein-related	5.56	73157	190	AT4G01870.1	3	5.52	0.0456	Q9SYI5	149.6	449.7	3.0054	0.0019	Q9SYI5_ARATH	Putative uncharacterized protein AT4g01870 (Putative uncharacterized protein At4g01870) (Putative uncharacterized protein T7B11.13) (TolB-related protein)
PGM2 Phosphoglucomutase/phosphomannomutase family protein	5.45	63710	1348	AT1G70730.1	13	29.06	0.003	Q9SGC1	154.3	433.3	2.8078	0.0041	PGMC2_ARATH	Probable phosphoglucomutase, cytoplasmic 2 (PGM 2) (EC 5.4.2.2) (Glucose phosphomutase 2)
PDS3, PDS, PDE226 phytoene desaturase 3	6.05	63420	336	AT4G14210.1	11	17.67	0.0161	Q07356	191.6	537.2	2.8047	0.0022	PDS_ARATH	15-cis-phytoene desaturase, chloroplastic/chromoplastic (EC 1.3.5.5) (Phytoene dehydrogenase)
BTF3, ATBTF3 basic transcription factor 3	7.62	17946	8235	AT1G17880.1	8	58.79	0	Q9SMW7	60.6	170.0	2.8033	0.0079	BTF3_ARATH	Basic transcription factor 3 (AtBTF3) (Nascent polypeptide-associated complex subunit beta)
ACO3 aconitase 3	6.74	108829	1016	AT2G05710.1	18	17.98	0.0036	Q9SIB9	913.7	2548.1	2.7889	0.0041	ACO2M_ARATH	Aconitate hydratase 2, mitochondrial (Aconitase 2) (EC 4.2.1.3) (Citrate hydro-lyase 2)
6-phosphogluconate dehydrogenase family protein	5.16	53720	1555	AT1G64190.1	12	24.44	0.0019	Q9SH69	174.9	486.4	2.7813	0.0078	6PGD1_ARATH	6-phosphogluconate dehydrogenase, decarboxylating 1, chloroplastic (EC 1.1.1.44)
Ribosomal L29 family protein	11.37	14285	5488	AT3G09500.1	11	35.77	0	Q9SF53	131.7	364.6	2.7691	0.0044	RL351_ARATH	60S ribosomal protein L35-1
Aldolase-type TIM barrel family protein	6.02	54394	280	AT1G16350.1	16	31.67	0.0196	Q9SA34	204.5	563.1	2.7539	0.0035	IMDH2_ARATH	Inosine-5'-monophosphate dehydrogenase 2 (IMP dehydrogenase 2) (IMPD 2) (IMPDH 2) (EC 1.1.1.205)
SNX1, ATSNX1 sorting nexin 1	6.87	46637	258	AT5G06140.1	13	31.09	0.0244	Q9FG38	147.0	398.3	2.7091	0.0033	SNX1_ARATH	Sorting nexin 1 (AtSNX1) (Vacuolar protein sorting-associated protein 5 homolog)
ATPMEPCRA, PMEPCRA methylesterase PCR A	8.97	62144	2208	AT1G11580.1	16	26.21	0	Q1JPL7	318.8	862.9	2.7063	0.0073	PME18_ARATH	Pectinesterase/pectinesterase inhibitor 18 (AtPMEpcrA) [Cleaved into: Pectinesterase inhibitor 18 (Pectin methylesterase inhibitor 18); Bifunctional pectinesterase 18/rRNA N-glycosylase (PE 18) (EC 3.1.1.11) (EC 3.2.2.22) (Pectin methylesterase 18) (Pectin methylesterase 4) (AtPME4) (Ribosome-inactivating protein)]
EMB86, EMB1030, EMB263 Alanyl-tRNA synthetase, class IIc	5.27	108287	386	AT5G22800.1	36	36.61	0.0118	Q9FFC7	1693.9	1168.5	0.6898	0.0071	SYAP_ARATH	Alanine--tRNA ligase, chloroplastic/mitochondrial (EC 6.1.1.7) (Alanyl-tRNA synthetase) (AlaRS) (Protein EMBRYO DEFECTIVE 1030) (Protein EMBRYO DEFECTIVE 263) (Protein EMBRYO DEFECTIVE 86)
SHD, HSP90.7, AtHsp90.7, AtHsp90-7 Chaperone protein htpG family protein	4.75	94261	829	AT4G24190.1	38	37.06	0.0036	Q9STX5	7933.7	5431.2	0.6846	0.0004	ENPL_ARATH	Endoplasmin homolog (Glucose-regulated protein 94 homolog) (GRP-94 homolog) (Heat shock protein 90-7) (AtHSP90.7) (AtHsp90-7) (Protein SHEPHERD)
Glycosyl hydrolase family protein	9.36	68497	2229	AT5G20950.1	21	29.49	0	Q8W112	7328.4	4635.2	0.6325	0.0014	Q8W112_ARATH	Beta-D-glucan exohydrolase-like protein (Glycosyl hydrolase family protein) (Putative beta-D-glucan exohydrolase)
GTP binding Elongation factor Tu family protein	9.46	49845	5156	AT1G07920.1	39	42.32	0	Q0WL56	8770.0	5167.0	0.5892	0.0018	EF1A3_ARATH	Elongation factor 1-alpha 3 (EF-1-alpha 3) (eEF-1A3)
ACO2, ATACO2 ACC oxidase 2	4.80	36411	7256	AT1G62380.1	9	14.69	0	Q41931	3426.3	1997.3	0.5829	0.0010	ACCO2_ARATH	1-aminocyclopropane-1-carboxylate oxidase 2 (ACC oxidase 2) (AtACO2) (EC 1.14.17.4)
TGG1, BGLU38 thioglucoside glucohydrolase 1	5.46	51994	1301	AT5G26000.2	23	45.61	0.003	P37702	9025.5	5054.7	0.5600	0.0002	BGL38_ARATH	Myrosinase 1 (EC 3.2.1.147) (Beta-glucosidase 38) (AtBGLU38) (EC 3.2.1.21) (Sinigrinase 1) (Thioglucosidase 1)
ACT domain-containing small subunit of acetolactate synthase protein	9.30	54101	2418	AT2G31810.1	17	37.47	0	Q93YZ7	1269.3	701.4	0.5526	0.0086	ILVH2_ARATH	Acetolactate synthase small subunit 2, chloroplastic (Acetohydroxy-acid synthase small subunit) (AHAS) (ALS)
ATPDIL2-1, UNE5, MEE30, PDI11, ATPDI11 thioredoxin family protein	5.70	39839	868	AT2G47470.1	29	50.14	0.0036	O22263	1336.3	672.6	0.5033	0.0057	PDI21_ARATH	Protein disulfide-isomerase like 2-1 (AtPDIL2-1) (EC 5.3.4.1) (P5) (Protein MATERNAL EFFECT EMBRYO ARREST 30) (Protein UNFERTILIZED EMBRYO SAC 5) (Protein disulfide isomerase 11) (AtPDI11) (Protein disulfide-isomerase A6) (Protein disulfide-isomerase like 4-1) (AtPDIL4-1)
UBC28 ubiquitin-conjugating enzyme 28	7.99	16681	1442	AT1G64230.1	3	20.27	0.003	Q94F47	349.8	168.5	0.4818	0.0049	UBC28_ARATH	Ubiquitin-conjugating enzyme E2 28 (EC 2.3.2.23) (E2 ubiquitin-conjugating enzyme 28) (Ubiquitin carrier protein 28)
ATIMD2, IMD2 isopropylmalate dehydrogenase 2	5.69	43542	786	AT1G80560.1	5	16.54	0.0036	P93832	131.0	62.4	0.4767	0.0035	LEU32_ARATH	3-isopropylmalate dehydrogenase 2, chloroplastic (3-IPM-DH 2) (IMDH 2) (EC 1.1.1.85) (Beta-IPM dehydrogenase 2)
Thioredoxin family protein	9.11	37325	1126	AT5G03880.1	4	24.78	0.003	Q940I2	250.7	100.0	0.3990	0.0030	Q940I2_ARATH	Putative uncharacterized protein MED24.18 (Thioredoxin family protein)
Hyaluronan / mRNA binding family	9.25	37999	9496	**AT5G47210.1**	19	48.18	0	A8MQD7	2831.1	1040.3	0.3675	0.0009	A8MQD7_ARATH	Hyaluronan / mRNA binding family protein
OASA1, OLD3, CYTACS1 O-acetylserine (thiol) lyase (OAS-TL) isoform A1	5.81	33919	7592	AT4G14880.1	27	47.52	0	P47998	1329.3	487.9	0.3670	0.0100	CYSK1_ARATH	Cysteine synthase 1 (EC 2.5.1.47) (At.OAS.5-8) (Beta-substituted Ala synthase 1;1) (ARAth-Bsas1;1) (CSase A) (AtCS-A) (Cys-3A) (O-acetylserine (thiol)-lyase 1) (OAS-TL A) (O-acetylserine sulfhydrylase) (Protein ONSET OF LEAF DEATH 3)
BXL2, ATBXL2 beta-xylosidase 2	8.27	83897	478	**AT1G02640.1**	7	12.24	0.0105	Q94KD8	630.0	219.9	0.3491	0.0058	BXL2_ARATH	Probable beta-D-xylosidase 2 (AtBXL2) (EC 3.2.1.-)
Protein of unknown function (DUF498/DUF598)	8.80	19171	546	AT2G44525.1	2	16.47	0.0053	Q8RUX8	117.8	39.0	0.3315	0.0052	Q8RUX8_ARATH	At2g44525 (Expressed protein) (Uncharacterized protein)

**Protein Description (24 hours)**	IEP	mw	max score	accession	reported peptides	sequence coverage	FDR level	UniProt	AVE A24	AVE C24	A24/C24 Avg	24 p-value	Protein names	Protein names (UniProt)
VDAC2, ATVDAC2 voltage dependent anion channel 2	9.22	29652	3403	AT5G67500.1	15	42.75	0	Q9FJX3	345.3	73.0	4.7309	0.0013	VDAC2_ARATH	Mitochondrial outer membrane protein porin 2 (Voltage-dependent anion-selective channel protein 2) (AtVDAC2) (VDAC-2)
ubiquitin family protein	4.63	45500	210	AT3G13235.3	5	9.93	0.034	F4JC86	599.0	127.0	4.7157	0.0088	F4JC86_ARATH	DNA damage-inducible protein 1
ACC1, AT-ACC1, EMB22, GK, PAS3 acetyl-CoA carboxylase 1	6.07	252979	358	AT1G36160.1	31	16.1	0.0145	Q38970	8630.1	1840.8	4.6882	0.0004	ACC1_ARATH	Acetyl-CoA carboxylase 1 (AtACC1) (EC 6.4.1.2) (Protein EMBRYO DEFECTIVE 22) (Protein GURKE) (Protein PASTICCINO 3) [Includes: Biotin carboxylase (EC 6.3.4.14)]
Ribosomal protein L13 family protein	10.83	23694	547	AT3G07110.1	7	20.87	0.0053	Q9SFU1	213.7	48.0	4.4493	0.0030	R13A1_ARATH	60S ribosomal protein L13a-1
ATPRX Q Thioredoxin superfamily protein	9.86	23849	9344	AT3G26060.1	12	39.81	0	Q9LU86	440.0	108.5	4.0571	0.0000	PRXQ_ARATH	Peroxiredoxin Q, chloroplastic (EC 1.11.1.15) (Thioredoxin reductase)
Ribosomal protein L10 family protein	4.57	30675	9150	AT3G09200.2	12	27.18	0	Q42112	437.1	119.0	3.6721	0.0001	RLA02_ARATH	60S acidic ribosomal protein P0-2
Acyl-CoA N-acyltransferases (NAT) superfamily protein	7.01	22057	1996	AT5G13780.1	8	53.13	0	Q9FKI4	672.2	188.8	3.5611	0.0074	Q9FKI4_ARATH	Peptide alpha-N-acetyltransferase (Putative uncharacterized protein MXE10.5) (Silencing group B protein) (Similarity to N-acetyltransferase)
SBE2.2 starch branching enzyme 2.2	5.45	92991	1842	AT5G03650.1	15	21.99	0.0019	Q9LZS3	1052.8	300.3	3.5062	0.0011	GLGB2_ARATH	1,4-alpha-glucan-branching enzyme 2-2, chloroplastic/amyloplastic (AtSBE II-2) (EC 2.4.1.18) (Branching enzyme 2) (AtBE2) (Starch-branching enzyme 2-2)
H2B, HTB2 histone B2	10.52	15732	5690	AT5G22880.1	6	17.93	0	Q9FFC0	192.3	54.9	3.5036	0.0004	H2B10_ARATH	Histone H2B.10 (HTB2)
Calcium-binding EF-hand family protein	4.58	16517	7647	AT1G12310.1	9	33.11	0	Q94AZ4	275.5	80.2	3.4365	0.0000	CML13_ARATH	Probable calcium-binding protein CML13 (Calmodulin-like protein 13)
Polyketide cyclase/dehydrase and lipid transport superfamily protein	5.9	17632	634	AT4G23670.1	10	24.5	0.0036	Q9SUR0	307.3	90.2	3.4070	0.0019	Q9SUR0_ARATH	AT4G23670 protein (AT4g23670/F9D16_140) (Polyketide cyclase/dehydrase and lipid transport superfamily protein) (Putative major latex protein)
transducin family protein / WD-40 repeat family protein	5.52	66840	373	AT3G18060.1	17	24.96	0.0133	Q9LV35	1094.8	343.4	3.1885	0.0045	AIP12_ARATH	Actin-interacting protein 1-2
PapD-like superfamily protein	9.8	33021	339	AT4G21450.1	5	16.61	0.0161	Q8VYN2	317.6	100.9	3.1484	0.0066	VAP42_ARATH	Vesicle-associated protein 4-2 (Plant VAP homolog 4-2) (AtPVA42) (VAMP-associated protein 4-2)
ATMDAR2 Pyridine nucleotide-disulphide oxidoreductase family protein	5.06	47537	2718	AT5G03630.1	20	42.53	0	Q93WJ8	1612.7	516.0	3.1251	0.0056	MDAR2_ARATH	Monodehydroascorbate reductase 2 (AtMDAR2) (EC 1.6.5.4)
Rieske (2Fe-2S) domain-containing protein	9.01	31898	5151	AT1G71500.1	17	41.11	0	Q9C9I7	449.6	148.9	3.0199	0.0004	Q9C9I7_ARATH	Putative uncharacterized protein At1g71500 (Putative uncharacterized protein At1g71500/F26A9_12) (Putative uncharacterized protein F26A9.12) (Rieske (2Fe-2S) domain-containing protein)
RPL12-C ribosomal protein L12-C	5.37	19682	6895	AT3G27850.1	16	42.78	0	P36212	490.3	162.5	3.0177	0.0090	RK123_ARATH	50S ribosomal protein L12-3, chloroplastic (CL12-C)
PETB photosynthetic electron transfer B	9.03	24267	2700	ATCG00720.1	4	15.35	0	P56773	293.9	101.0	2.9096	0.0012	CYB6_ARATH	Cytochrome b6
NIT3, AtNIT3 nitrilase 3	5.55	38364	1516	AT3G44320.1	8	13.58	0.0019	P46010	215.3	75.7	2.8463	0.0063	NRL3_ARATH	Nitrilase 3 (EC 3.5.5.1)

RNA polymerase I-associated factor PAF67	5.84	60696	260	AT5G25754.1	9	17.9	0.0239	Q8H152	348.3	123.6	2.8188	0.0088	Q8H152_ARATH	Eukaryotic translation initiation factor 3 subunit L (eIF3l)
SNX1, ATSNX1 sorting nexin 1	6.87	46637	258	AT5G06140.1	13	31.09	0.0244	Q9FG38	393.4	141.0	2.7909	0.0011	SNX1_ARATH	Sorting nexin 1 (AtSNX1) (Vacuolar protein sorting-associated protein 5 homolog)
emb1624 Translation initiation factor IF6	4.43	26881	1460	AT3G55620.1	3	9.39	0.003	Q9M060	138.8	51.1	2.7140	0.0001	IF62_ARATH	Eukaryotic translation initiation factor 6-2 (AteIF-6;2)
ATLFNR1, FNR1 ferredoxin-NADP(+)-oxidoreductase 1	8.21	40669	6052	AT5G66190.1	29	50.56	0	Q9FKW6	1737.1	641.7	2.7070	0.0048	FNRL1_ARATH	Ferredoxin--NADP reductase, leaf isozyme 1, chloroplastic (EC 1.18.1.2) (Leaf FNR 1) (AtLFNR1) (FNR-1)
UVR8 Regulator of chromosome condensation (RCC1) family protein	5.33	47518	811	AT5G63860.1	10	31.59	0.0036	Q9FN03	397.4	147.8	2.6883	0.0013	UVR8_ARATH	Ultraviolet-B receptor UVR8 (Protein UV-B RESISTANCE 8) (RCC1 domain-containing protein UVR8)
RPS11 ribosomal protein S11	12.65	15138	4477	ATCG00750.1	5	17.39	0	P56802	122.0	45.7	2.6663	0.0010	RR11_ARATH	30S ribosomal protein S11, chloroplastic
ATPF ATPase, F0 complex, subunit B/B', bacterial/chloroplast	8.61	21114	1418	ATCG00130.1	17	44.02	0.003	P56759	940.6	360.4	2.6101	0.0094	ATPF_ARATH	ATP synthase subunit b, chloroplastic (ATP synthase F(0) sector subunit b) (ATPase subunit I)
Ribosomal protein L7Ae/L30e/S12e/Gadd45 family protein	5.24	15719	4433	AT1G15930.1	6	40.28	0	Q9S9P1	146.6	56.5	2.5928	0.0009	RS121_ARATH	40S ribosomal protein S12-1
ACO3 aconitase 3	6.74	108829	1016	AT2G05710.1	18	17.98	0.0036	Q9SIB9	2029.7	787.9	2.5760	0.0008	ACO2M_ARATH	Aconitate hydratase 2, mitochondrial (Aconitase 2) (EC 4.2.1.3) (Citrate hydro-lyase 2)
FKBP-type peptidyl-prolyl cis-trans isomerase, putative	9.99	20149	978	AT3G12345.1	5	26.88	0.0036	Q9LHH3	110.0	42.8	2.5691	0.0064	Q9LHH3_ARATH	FKBP-type peptidyl-prolyl cis-trans isomerase, putative (Putative uncharacterized protein At3g12340) (Stress-enhanced protein 4) (Uncharacterized protein)
ATPAP26, PAP26 purple acid phosphatase 26	6.87	55067	391	AT5G34850.1	12	23.58	0.0118	Q949Y3	588.3	234.0	2.5144	0.0085	PPA26_ARATH	Bifunctional purple acid phosphatase 26 [Includes: Acid phosphatase (EC 3.1.3.2); Peroxidase (EC 1.11.1.7)]
MMZ3, UEV1C MMS ZWEI homologue 3	6.61	16496	1129	AT2G36060.3	5	29.86	0.003	Q9SJ44	84.5	33.9	2.4928	0.0040	UEV1C_ARATH	Ubiquitin-conjugating enzyme E2 variant 1C (Ubc enzyme variant 1C) (Protein MMS ZWEI HOMOLOG 3)
Ribosomal L5P family protein	10.43	28458	579	AT4G01310.1	20	59.54	0.0045	O04603	184.3	74.1	2.4882	0.0066	RK5_ARATH	50S ribosomal protein L5, chloroplastic
PHT3;1 phosphate transporter 3;1	9.45	40489	1538	AT5G14040.1	15	27.47	0.0019	Q9FMU6	400.9	162.1	2.4732	0.0001	MPCP3_ARATH	Mitochondrial phosphate carrier protein 3, mitochondrial (Mitochondrial phosphate transporter 3) (MPT3) (Phosphate transporter 3;1)
Bifunctional inhibitor/lipid-transfer protein/seed storage 2S albumin superfamily protein	9.76	30390	549	AT2G10940.1	3	6.87	0.0053	Q9SKI0	216.4	87.7	2.4678	0.0090	Q9SKI0_ARATH	At2g10940/F15K19.1 (Bifunctional inhibitor/lipid-transfer protein/seed storage 2S albumin superfamily protein) (Expressed protein) (Putative uncharacterized protein At2g10940; F15K19.1)
Ribosomal L29 family protein	11.37	14285	5488	AT3G09500.1	11	35.77	0	Q9SF53	298.5	121.0	2.4674	0.0001	RL351_ARATH	60S ribosomal protein L35-1
Ribosomal protein S26e family protein	11.49	15183	9214	AT2G40510.1	5	18.05	0	Q8LPJ7	199.4	81.6	2.4418	0.0034	RS262_ARATH	40S ribosomal protein S26-2
MAC3A MOS4-associated complex 3A	6.16	57265	242	AT1G04510.1	6	10.71	0.0253	Q94BR4	653.7	268.8	2.4321	0.0047	PR19A_ARATH	Pre-mRNA-processing factor 19 homolog 1 (EC 6.3.2.-) (MOS4-associated complex protein 3A) (MAC protein 3A) (Plant U-box protein 59) (U-box domain-containing protein 59)
NIT1, ATNIT1, NITI nitrilase 1	5.82	38551	2164	AT3G44310.1	9	13.58	0	P32961	351.1	145.3	2.4155	0.0016	NRL1_ARATH	Nitrilase 1 (EC 3.5.5.1)
Protein phosphatase 2C family protein	8.02	31190	679	AT4G28400.1	3	14.49	0.0036	Q93YW5	60.7	25.7	2.3609	0.0099	P2C58_ARATH	Probable protein phosphatase 2C 58 (AtPP2C58) (EC 3.1.3.16)
ATGSKB6, GLN1.3, GLN1;3 glutamine synthetase 1.3	5.65	38823	876	AT3G17820.1	7	6.78	0.0036	Q9LVI8	878.2	384.2	2.2856	0.0077	GLN13_ARATH	Glutamine synthetase cytosolic isozyme 1-3 (GS1) (EC 6.3.1.2) (Glutamate--ammonia ligase GLN1;3) (GLN1;3)
ATGSTF10, ERD13, ATGSTF4, GSTF10 glutathione S-transferase PHI 10	5.35	24230	4115	AT2G30870.1	11	27.91	0	P42761	339.4	148.8	2.2812	0.0012	GSTFA_ARATH	Glutathione S-transferase F10 (AtGSTF10) (EC 2.5.1.18) (AtGSTF4) (GST class-phi member 10) (Protein EARLY RESPONSE TO DEHYDRATION 13)
GRF10, 14-3-3EPSILON, GF14 EPSILON general regulatory factor 10	4.53	29029	3163	AT1G22300.1	13	48.43	0	P48347	563.3	249.9	2.2544	0.0011	14310_ARATH	14-3-3-like protein GF14 epsilon (General regulatory factor 10)
PSBE photosystem II reaction center protein E	4.63	9397	1010	ATCG00580.1	3	10.84	0.0036	P56779	238.6	106.0	2.2507	0.0090	PSBE_ARATH	Cytochrome b559 subunit alpha (PSII reaction center subunit V)
TAPX thylakoidal ascorbate peroxidase	6.91	46320	526	AT1G77490.1	11	28.17	0.0053	Q42593	286.4	127.6	2.2451	0.0047	APXT_ARATH	L-ascorbate peroxidase T, chloroplastic (EC 1.11.1.11) (Thylakoid-bound ascorbate peroxidase) (AtAPx06) (tAPX)
Coatomer, alpha subunit	6.45	137494	492	AT2G21390.1	16	14.61	0.0082	Q9SJT9	1237.8	554.2	2.2334	0.0002	COPA2_ARATH	Coatomer subunit alpha-2 (Alpha-coat protein 2) (Alpha-COP 2)
CAB3, AB180, LHCB1.2 chlorophyll A/B binding protein 3	5.12	28284	5528	AT1G29910.1	7	17.23	0	Q8VZ87	901.9	409.3	2.2036	0.0080	CB1B_ARATH	Chlorophyll a-b binding protein 3, chloroplastic (Chlorophyll a-b protein 180) (CAB-180) (LHCII type I CAB-3)
RCA rubisco activase	5.76	52381	14066	AT2G39730.1	59	65.61	0	P10896	8803.7	4008.1	2.1965	0.0040	RCA_ARATH	Ribulose bisphosphate carboxylase/oxygenase activase, chloroplastic (RA) (RuBisCO activase)
Lipase/lipooxygenase, PLAT/LH2 family protein	4.95	20415	1196	AT2G22170.1	5	20.22	0.003	Q9SIE7	128.4	58.5	2.1937	0.0036	Q9SIE7_ARATH	Expressed protein (PLAT-plant-stress domain-containing protein) (Putative uncharacterized protein At2g22170)
ATBBC1, BBC1, RSU2 breast basic conserved 1	11.46	23767	4950	AT3G49010.1	7	22.33	0	P41127	237.1	108.7	2.1810	0.0063	RL131_ARATH	60S ribosomal protein L13-1 (Protein BBC1 homolog)
Uncharacterized protein	9.6	5976	2882	AT2G40765.1	5	43.86	0	Q94K78	31.6	14.5	2.1772	0.0081	Q94K78_ARATH	Putative uncharacterized protein At2g40765 (Ubiquinol--cytochrome-c reductase) (Uncharacterized protein)
GS2, GLN2, ATGSL1 glutamine synthetase 2	6.44	47810	3875	AT5G35630.1	28	36.98	0	Q43127	2817.3	1296.5	2.1730	0.0001	GLNA2_ARATH	Glutamine synthetase, chloroplastic/mitochondrial (EC 6.3.1.2) (GS2) (Glutamate--ammonia ligase)
Adenine nucleotide alpha hydrolases-like superfamily protein	5.44	21686	3176	AT3G11930.1	7	28.14	0	Q9SF06	208.3	96.6	2.1562	0.0010	Q9SF06_ARATH	AT3g11930/MEC18.3 (At3g11930/MEC18.3) (F26K24.22 protein) (MEC18.3/MEC18.3) (Uncharacterized protein) (Universal stress protein-like protein)
Plastid-lipid associated protein PAP / fibrillin family protein	10.11	27221	3038	AT3G26070.1	6	21.49	0	Q9LU85	305.8	142.5	2.1458	0.0011	PAP4_ARATH	Probable plastid-lipid-associated protein 4, chloroplastic (Fibrillin-3a) (Plastoglobulin 25) (AtPGL25)
ADL3, CF1, DRP2B, DL3 dynamin-like 3	9.41	100513	399	AT1G59610.1	19	27.28	0.0118	Q9LQ55	499.9	234.3	2.1338	0.0065	DRP2B_ARATH	Dynamin-2B (EC 3.6.5.5) (Dynamin-like protein 3) (Dynamin-related protein 2B)
Protein CURVATURE THYLAKOID 1A, chloroplastic	9.57	17755	8203	AT4G01150.1	10	56.71	0	O04616	177.2	83.9	2.1120	0.0003	CUT1A_ARATH	Protein CURVATURE THYLAKOID 1A, chloroplastic
PKP1, PKP-ALPHA Pyruvate kinase family protein	5.54	65644	751	AT3G22960.1	17	27.35	0.0036	Q9LIK0	716.9	343.1	2.0896	0.0008	PKP1_ARATH	Plastidial pyruvate kinase 1, chloroplastic (PK1) (PKp1) (EC 2.7.1.40) (Pyruvate kinase II) (Pyruvate kinase isozyme A) (PKP-ALPHA)
PSB29, THF1 photosystem II reaction center PSB29 protein	9.43	34024	2738	AT2G20890.1	12	29.33	0	Q9SKT0	693.4	332.0	2.0882	0.0028	THF1_ARATH	Protein THYLAKOID FORMATION 1, chloroplastic
ERD14 Dehydrin family protein	5.24	20786	1915	AT1G76180.1	10	40.54	0.0019	P42763	146.9	70.9	2.0711	0.0048	ERD14_ARATH	Dehydrin ERD14
HPR, ATHPR1 hydroxypyruvate reductase	6.77	42476	2130	AT1G68010.1	27	54.15	0	Q9C9W5	1086.0	529.6	2.0508	0.0012	HPR1_ARATH	Glycerate dehydrogenase HPR, peroxisomal (GDH) (EC 1.1.1.29) (NADH-dependent hydroxypyruvate reductase 1) (AtHPR1) (HPR 1)
PEX11D peroxin 11D	10.38	26172	874	AT2G45740.1	8	23.31	0.0036	O80845	106.3	52.4	2.0282	0.0082	PX11D_ARATH	Peroxisomal membrane protein 11D (Peroxin-11D) (AtPEX11d)
Aldolase-type TIM barrel family protein	9.55	38224	6919	AT3G14420.4	17	17.24	0	Q9LRR9	533.5	268.2	1.9890	0.0029	GLO1_ARATH	Peroxisomal (S)-2-hydroxy-acid oxidase GLO1 (EC 1.1.3.15) (Glycolate oxidase 1) (AtGLO1) (GOX 1) (Short chain alpha-hydroxy acid oxidase GLO1)
TUB5 tubulin beta-5 chain	4.47	50913	3057	AT1G20010.1	7	16.04	0	P29513	721.1	370.9	1.9443	0.0049	TBB5_ARATH	Tubulin beta-5 chain (Beta-5-tubulin)
Mannose-binding lectin superfamily protein	4.89	32023	3749	AT3G16450.1	12	50	0	O04311	322.2	166.5	1.9355	0.0095	JAL33_ARATH	Jacalin-related lectin 33
PSBD photosystem II reaction center protein D	5.36	39776	5017	ATCG00270.1	12	26.06	0	P56761	2790.4	1450.6	1.9237	0.0010	PSBD_ARATH	Photosystem II D2 protein (PSII D2 protein) (EC 1.10.3.9) (Photosystem Q(A) protein)
TUF, emb2448, TUFF, VHA-E1 vacuolar ATP synthase subunit E1	5.99	26288	1838	AT4G11150.1	21	62.17	0.0019	Q39258	198.1	104.0	1.9052	0.0094	VATE1_ARATH	V-type proton ATPase subunit E1 (V-ATPase subunit E1) (Protein EMBRYO DEFECTIVE 2448) (Vacuolar H(+)-ATPase subunit E isoform 1) (Vacuolar proton pump subunit E1)
ATRPL23A, RPL23A, RPL23AA ribosomal protein L23AA	10.64	17441	5459	AT2G39460.1	12	35.06	0	Q8LD46	483.3	254.5	1.8991	0.0048	R23A1_ARATH	60S ribosomal protein L23a-1 (AtRPL23A-1)
ATPD ATP synthase delta-subunit gene	9.56	25669	4833	AT4G09650.1	18	39.74	0	Q9SSS9	792.6	426.1	1.8603	0.0044	ATPD_ARATH	ATP synthase subunit delta, chloroplastic (ATP synthase F(1) sector subunit delta) (F-type ATPase subunit delta)
AGT, AGT1, SGAT alanine:glyoxylate aminotransferase	7.77	44493	1102	AT2G13360.1	16	29.68	0.003	Q56YA5	520.4	280.5	1.8550	0.0077	SGAT_ARATH	Serine--glyoxylate aminotransferase (EC 2.6.1.45) (Alanine--glyoxylate aminotransferase) (AGT) (EC 2.6.1.44) (Asparagine aminotransferase) (EC 2.6.1.-) (Serine--pyruvate aminotransferase) (EC 2.6.1.51)
EMB1080 Nucleic acid-binding, OB-fold-like protein	11	18185	2380	AT3G48930.1	8	15	0	P16181	166.6	90.1	1.8483	0.0011	RS111_ARATH	40S ribosomal protein S11-1 (Protein EMBRYO DEFECTIVE 1080)
CA1, ATBCA1, SABP3, ATSABP3 carbonic anhydrase 1	5.41	29846	14147	AT3G01500.1	12	20.74	0	P27140	2103.3	1150.5	1.8282	0.0035	BCA1_ARATH	Beta carbonic anhydrase 1, chloroplastic (AtbCA1) (AtbetaCA1) (EC 4.2.1.1) (Beta carbonate dehydratase 1) (Protein SALICYLIC ACID-BINDING PROTEIN 3) (AtSABP3)
PSAB Photosystem I, PsaA/PsaB protein	6.95	82590	1547	ATCG00340.1	10	9.81	0.0019	P56767	2402.7	1319.4	1.8211	0.0013	PSAB_ARATH	Photosystem I P700 chlorophyll a apoprotein A2 (EC 1.97.1.12) (PSI-B) (PsaB)
HIPL1 protein	5.13	76822	574	AT1G74790.1	8	12.37	0.0045	Q9SSG3	348.2	192.2	1.8117	0.0034	HIPL1_ARATH	HIPL1 protein
VAR1, FTSH5 FtsH extracellular protease family	5.19	75403	187	AT5G42270.1	13	16.76	0.0474	Q9FH02	438.8	243.2	1.8046	0.0072	FTSH5_ARATH	ATP-dependent zinc metalloprotease FTSH 5, chloroplastic (AtFTSH5) (EC 3.4.24.-) (Protein VARIEGATED 1)
MTHFR1 methylenetetrahydrofolate reductase 1	5.44	66859	2560	AT3G59970.3	19	28.21	0	Q9SE60	1052.1	583.3	1.8038	0.0008	MTHR1_ARATH	Methylenetetrahydrofolate reductase 1 (AtMTHFR1) (EC 1.5.1.20)
GRF9, GF14 MU general regulatory factor 9	4.64	29634	3041	AT2G42590.1	16	45.25	0	Q96299	506.6	282.3	1.7946	0.0029	14339_ARATH	14-3-3-like protein GF14 mu (General regulatory factor 9)
Ribosomal L27e protein family	10.58	15149	3826	AT4G15000.2	5	29.01	0	P51419	95.2	53.5	1.7797	0.0010	RL273_ARATH	60S ribosomal protein L27-3
LOX2, ATLOX2 lipoxygenase 2	5.29	102616	616	AT3G45140.1	27	22.32	0.0037	P38418	1086.8	617.0	1.7616	0.0077	LOX2_ARATH	Lipoxygenase 2, chloroplastic (AtLOX2) (EC 1.13.11.12)
HAP6 ribophorin II (RPN2) family protein	7.33	74953	988	AT4G21150.1	23	28.8	0.0036	Q93Z16	1783.8	1025.4	1.7396	0.0001	RPN2_ARATH	Dolichyl-diphosphooligosaccharide--protein glycosyltransferase subunit 2 (EC 2.4.99.18) (Protein HAPLESS 6) (Ribophorin II) (RPN-II) (Ribophorin-2)
PDF2 prefoldin 2	9.06	16583	5170	AT3G22480.2	6	38.51	0	Q9LJ98	80.8	46.9	1.7216	0.0092	PFD2_ARATH	Probable prefoldin subunit 2

ATPHB4, PHB4 prohibitin 4	7.77	30638	3343	AT3G27280.1	11	26.88	0	Q9LK25	364.6	216.4	1.6849	0.0008	PHB4_ARATH	Prohibitin-4, mitochondrial (Atphb4)
PSBO2, PSBO-2, OEC33 photosystem II subunit O-2	5.81	35247	12122	AT3G50820.1	27	71.3	0	Q9S841	1203.0	731.7	1.6442	0.0087	PSBO2_ARATH	Oxygen-evolving enhancer protein 1-2, chloroplastic (OEE1) (33 kDa subunit of oxygen evolving system of photosystem II) (33 kDa thylakoid membrane protein) (Manganese-stabilizing protein 2) (MSP-2) (OEC 33 kDa subunit)
LHCB4.1 light harvesting complex photosystem II	5.7	31197	5332	AT5G01530.1	15	34.48	0	Q07473	1039.9	635.4	1.6366	0.0046	CB4A_ARATH	Chlorophyll a-b binding protein CP29.1, chloroplastic (LHCB4.1) (LHCII protein 4.1)
RBB1, REGULATOR OF BULB BIOGENESIS1	4.04	323634	215	AT5G40450.1	29	13.85	0.033	Q9FND5	4944.3	3050.6	1.6208	0.0085	Q9FND5_ARATH	Similarity to heat shock protein
ATHM4, TRX-M4, ATM4 thioredoxin M-type 4	9.99	21343	3017	AT3G15360.1	13	35.75	0	Q9SEU6	607.2	375.9	1.6152	0.0064	TRXM4_ARATH	Thioredoxin M4, chloroplastic (AtTrxm4)
MD-2-related lipid recognition domain-containing protein	8.28	16298	7969	AT3G44100.1	5	33.55	0	Q9LXQ2	180.3	114.1	1.5804	0.0011	Q9LXQ2_ARATH	At3g44100 (MD-2-related lipid recognition domain-containing protein) (Putative uncharacterized protein At3g44100) (Putative uncharacterized protein F26G5_50)
AAC1 ADP/ATP carrier 1	10.13	41590	4832	AT3G08580.1	15	18.11	0	P31167	1483.4	941.6	1.5754	0.0002	ADT1_ARATH	ADP,ATP carrier protein 1, mitochondrial (ADP/ATP translocase 1) (Adenine nucleotide translocator 1) (ANT 1)
JR1 Mannose-binding lectin superfamily protein	5.11	32147	6082	AT3G16470.3	11	33.67	0	O04309	386.2	248.0	1.5572	0.0061	JAL35_ARATH	Jacalin-related lectin 35 (JA-responsive protein 1) (Myrosinase-binding protein-like At3g16470)
Zincin-like metalloproteases family protein	5.31	79272	1305	AT5G10540.1	10	10.56	0.003	Q949P2	328.6	211.6	1.5530	0.0041	COPDA_ARATH	Probable cytosolic oligopeptidase A (EC 3.4.24.70) (Thimet metalloendopeptidase 2) (Zincin-like metalloproteases family protein 2)
ATLFNR2 ferredoxin-NADP(+)-oxidoreductase 2	8.61	39575	1891	AT1G20020.2	22	47.14	0.0019	Q8W493	769.6	495.6	1.5530	0.0059	FNRL2_ARATH	Ferredoxin--NADP reductase, leaf isozyme 2, chloroplastic (EC 1.18.1.2) (Leaf FNR 2) (AtLFNR2) (FNR-2)
SHM1, STM, SHMT1 serine transhydroxymethyltransferase 1	8.28	57572	4902	AT4G37930.1	42	54.16	0	Q9SZJ5	3239.3	2167.4	1.4945	0.0098	GLYM1_ARATH	Serine hydroxymethyltransferase 1, mitochondrial (AtSHMT1) (EC 2.1.2.1) (Glycine hydroxymethyltransferase 1) (Serine Transhydroxymethyltransferase) (STM) (Serine methylase 1)
NAD(P)-linked oxidoreductase superfamily protein	8.69	46712	983	AT1G04420.1	13	27.43	0.0036	Q8VZ23	306.7	207.8	1.4759	0.0073	Q8VZ23_ARATH	NAD(P)-linked oxidoreductase superfamily protein (Putative uncharacterized protein At1g04420)
Insulinase (Peptidase family M16) protein	5.9	54573	1780	AT1G51980.1	18	33.4	0.0019	Q9ZU25	393.0	272.0	1.4451	0.0030	MPPA1_ARATH	Probable mitochondrial-processing peptidase subunit alpha-1, mitochondrial (EC 3.4.24.64) (Alpha-MPP 1)
HAD superfamily, subfamily IIIB acid phosphatase	7.9	31456	1719	AT5G44020.1	20	47.79	0.0019	Q9FNC4	269.4	187.4	1.4375	0.0021	Q9FNC4_ARATH	HAD superfamily, subfamily IIIB acid phosphatase (Putative vegetative storage protein) (Vegetative storage protein-like)
Cystathionine beta-synthase (CBS) family protein	9.41	22843	1894	AT5G10860.1	13	36.89	0.0019	Q9LEV3	125.7	183.2	0.6858	0.0015	CBSX3_ARATH	CBS domain-containing protein CBSX3, mitochondrial
ROC4 rotamase CYP 4	8.72	28550	3282	AT3G62030.1	19	56.92	0	P34791	758.1	1125.9	0.6733	0.0002	CP20C_ARATH	Peptidyl-prolyl cis-trans isomerase CYP20-3, chloroplastic (PPIase CYP20-3) (EC 5.2.1.8) (Cyclophilin of 20 kDa 3) (Cyclosporin A-binding protein) (Rotamase CYP20-3) (Rotamase cyclophilin-4)
Translation elongation factor EF1B, gamma chain	5.4	46629	1864	AT1G57720.1	25	32.93	0.0019	Q9FVT2	586.1	913.8	0.6413	0.0093	EF1G2_ARATH	Probable elongation factor 1-gamma 2 (EF-1-gamma 2) (eEF-1B gamma 2)
Nuclear pore complex protein NUP54	6.25	41817	615	AT1G24310.1	3	9.28	0.0037	Q8GYF7	40.0	63.7	0.6286	0.0071	NUP54_ARATH	Nuclear pore complex protein NUP54 (Nucleoporin 54)
phenylalanyl-tRNA synthetase class IIc family protein	5.96	49651	1938	AT3G58140.1	18	39.39	0.0019	Q94K73	1958.0	3125.6	0.6264	0.0037	SYFM_ARATH	Phenylalanine--tRNA ligase, chloroplastic/mitochondrial (EC 6.1.1.20) (Phenylalanyl-tRNA synthetase) (PheRS)
Fumarylacetoacetate (FAA) hydrolase family	8.99	24474	3168	AT3G16700.1	4	27.23	0	Q9LUR3	152.4	250.6	0.6079	0.0069	Q9LUR3_ARATH	Isomerase-like protein (Putative decarboxilase) (Putative fumarylacetoacetate hydrolase)
Cyclophilin-like peptidyl-prolyl cis-trans isomerase family protein	7.06	30790	241	AT5G35100.1	3	13.52	0.0253	O65220	48.0	80.0	0.6002	0.0029	CPY28_ARATH	Peptidyl-prolyl cis-trans isomerase CYP28, chloroplastic (PPIase CYP28) (EC 5.2.1.8) (Cyclophilin of 28 kDa) (Cyclophilin-28)
Hyaluronan / mRNA binding family	9.25	37999	9496	AT5G47210.1	19	48.18	0	Q9LVT8	1483.6	2490.9	0.5956	0.0012	Q9LVT8_ARATH	At5g47210 (Hyaluronan / mRNA binding family protein) (Putative uncharacterized protein At5g47210)
ATCS, CSY4 Citrate synthase family protein	6.43	52997	457	AT2G44350.1	15	37.84	0.0111	P20115	464.4	863.8	0.5377	0.0027	CISY4_ARATH	Citrate synthase 4, mitochondrial (EC 2.3.3.16)
Uncharacterized protein	9.03	34526	197	AT1G19140.2	5	22.44	0.0401	Q9LMB2	26.1	51.8	0.5033	0.0099	Q9LMB2_ARATH	T29M8.1 protein (Uncharacterized protein)
Ribosomal protein L6 family	9.94	22088	1217	AT4G10450.1	5	21.65	0.003	Q9SZX9	32.1	65.8	0.4882	0.0063	RL92_ARATH	60S ribosomal protein L9-2
ENOC cytosolic enolase	5.18	52113	289	AT2G29560.1	7	21.89	0.0191	Q9ZW34	298.3	622.8	0.4790	0.0016	ENO3_ARATH	Cytosolic enolase 3 (EC 4.2.1.11) (2-phospho-D-glycerate hydro-lyase 3) (2-phosphoglycerate dehydratase 3)
sks5 SKU5 similar 5	8.53	60212	2647	AT1G76160.1	10	18.67	0	Q9SGR6	659.0	1389.0	0.4744	0.0037	Q9SGR6_ARATH	AT1G76160 protein (SKU5-like 5 protein) (T23E18.10)
ROC3 rotamase CYP 3	8.6	18720	1954	AT2G16600.1	5	28.9	0.0019	Q38900	99.9	211.6	0.4721	0.0010	CP19A_ARATH	Peptidyl-prolyl cis-trans isomerase CYP19-1 (PPIase CYP19-1) (EC 5.2.1.8) (Cyclophilin of 19 kDa 1) (Rotamase cyclophilin-3)
BXL2, ATBXL2 beta-xylosidase 2	8.27	83897	478	AT1G02640.1	7	12.24	0.0105	Q94KD8	324.0	805.9	0.4020	0.0036	BXL2_ARATH	Probable beta-D-xylosidase 2 (AtBXL2) (EC 3.2.1.-)
Uncharacterized protein	6.25	22597	806	AT5G66090.1	6	33.81	0.0036	Q9FKX3	42.9	109.7	0.3916	0.0045	Q9FKX3_ARATH	Uncharacterized protein
RNA recognition motif (RRM)-containing protein	6.56	39180	196	AT1G79880.2	4	17.1	0.041	Q0V7U7	662.6	1715.8	0.3862	0.0063	LA2_ARATH	La protein 2 (AtLa2)
LTP5 lipid transfer protein 5	10.59	13008	1205	AT3G51600.1	2	6.78	0.003	Q9XFS7	32.0	93.4	0.3431	0.0068	NLTP5_ARATH	Non-specific lipid-transfer protein 5 (LTP 5)
BCE2, LTA1, DIN3 2-oxoacid dehydrogenases acyltransferase family protein	6.3	53106	299	AT3G06850.1	5	19.25	0.0176	Q9M7Z1	64.7	235.7	0.2743	0.0023	ODB2_ARATH	Lipoamide acyltransferase component of branched-chain alpha-keto acid dehydrogenase complex, mitochondrial (EC 2.3.1.168) (Branched-chain alpha-keto acid dehydrogenase complex component E2) (BCE2) (BCKAD-E2) (BCKADE2) (Dihydrolipoamide acetyltransferase component of branched-chain alpha-keto acid dehydrogenase complex) (Dihydrolipoamide branched chain transacylase) (Dihydrolipoyllysine-residue (2-methylpropanoyl)transferase) (Protein DARK INDUCIBLE 3)
Uncharacterized protein	10.87	7813	16444	AT5G24165.1	5	52	0	Q8LDQ8	45.7	187.7	0.2436	0.0032	Q8LDQ8_ARATH	At5g24165 (Putative uncharacterized protein At5g24165) (Uncharacterized protein)
SS2 strictosidine synthase 2	5.44	35578	305	AT1G74020.1	3	11.94	0.0176	P94111	41.1	350.5	0.1174	0.0080	SSL12_ARATH	Protein STRICTOSIDINE SYNTHASE-LIKE 12 (AtSSL12) (EC 4.3.3.2) (Strictosidine synthase 1) (SS-1) (Strictosidine synthase 13) (AtSS13)

Table S4. Proteins Identified and Matched to Cell Wall Proteins Based Upon Protein Interactions

Accession	UniProt (Gene)	Description	Reported peptides	Coverage
AT3G22845.1	Q9LIL4 (P24B3_ARATH)	p24 family protein beta3 (p24beta3)	3	9.81
AT4G35450.4	Q9SAR5 (AKR2A_ARATH)	Ankyrin repeat domain-containing protein 2A	4	21.71
AT1G04510.1	Q94BR4 (PR19A_ARATH)	Prp19A, MAC3A	6	10.71
AT2G47470.1	O22263 (PDI21_ARATH)	Protein disulfide-isomerase like 2-1	29	50.14
AT3G09440.1	O65719 (HSP7C_ARATH)	Heat shock 70kDa protein 3	21	21.73

STRING Analyses of the proteins listed above.

Szklarczyk D, Franceschini A, Wyder S, Forslund K, Heller D, Huerta-Cepas J, Simonovic M, Roth A, Santos A, Tsafou KP, Kuhn M, Bork P, Jensen LJ, von Mering C. STRING v10: protein-protein interaction networks, integrated over the tree of life. Nucleic Acids Res. **43** [2015] D447-452.

Gene Neighborhood
Co-expression
Experimentally determined
Text-mining
From curated database
Protein homology
Gene co-occurence

AT3G22845 emp24/gp25L/p24 family/GOLD domain-containing protein; Involved in vesicular protein trafficking. Mainly functions in the early secretory pathway but also in post-Golgi membranes. Thought to act as cargo receptor at the lumenal side for incorporation of secretory cargo molecules into transport vesicles and to be involved in vesicle coat formation at the cytoplasmic side (By similarity) (214 aa)

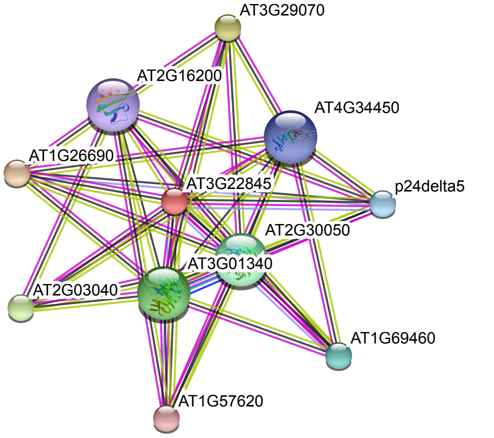

AT1G26690 emp24/gp25L/p24 family/GOLD domain-containing protein; Involved in vesicular protein trafficking. Mainly functions in the early secretory pathway. Thought to act as cargo receptor at the lumenal side for incorporation of secretory cargo molecules into transport vesicles and to be involved in vesicle coat formation at the cytoplasmic side (By similarity) (214 aa); **SCORE:** 0.999

AT3G29070 emp24/gp25L/p24 family/GOLD domain-containing protein; Involved in vesicular protein trafficking. Mainly functions in the early secretory pathway. Thought to act as cargo receptor at the lumenal side for incorporation of secretory cargo molecules into transport vesicles and to be involved in vesicle coat formation at the cytoplasmic side (By similarity) (225 aa); **SCORE:** 0.987

AT2G03040 emp24/gp25L/p24 family/GOLD domain-containing protein (166 aa); **SCORE:** 0.987

AT3G01340 transducin/WD-40 repeat-containing protein (302 aa); **SCORE:** 0.974

AT2G30050 protein transport protein SEC13 (302 aa); **SCORE:** 0.974

AT1G69460 emp24/gp25L/p24 family/GOLD domain-containing protein; Involved in vesicular protein trafficking. Mainly functions in the early secretory pathway. Thought to act as cargo receptor at the lumenal side for incorporation of secretory cargo molecules into transport vesicles and to be involved in vesicle coat formation at the cytoplasmic side (By similarity) (214 aa); **SCORE:** 0.952

p24delta5 p24 subfamily delta 5; Involved in vesicular protein trafficking. Mainly functions in the early secretory pathway. Thought to act as cargo receptor at the lumenal side for incorporation of secretory cargo molecules into transport vesicles and to be involved in vesicle coat formation at the cytoplasmic side (By similarity). Interacts with p24beta2 at endoplasmic reticulum export sites for endoplasmic reticulum exit and coupled transport to the Golgi apparatus. Once in the Golgi, interacts very efficiently with the COPI machinery for retrograde transport back to the endoplasmic reticulum (216 aa); **SCORE:** 0.944

AT4G34450 coatomer subunit gamma; The coatomer is a cytosolic protein complex that binds to dilysine motifs and reversibly associates with Golgi non- clathrin-coated vesicles, which further mediate biosynthetic protein transport from the ER, via the Golgi up to the trans Golgi network. Coatomer complex is required for budding from Golgi membranes, and is essential for the retrograde Golgi-to-ER transport of dilysine-tagged proteins (By similarity) (886 aa); **SCORE:** 0.931

AT2G16200 coatomer gamma subunit appendage domain-containing protein (83 aa); **SCORE:** 0.931

AT1G57620 emp24/gp25L/p24 family/GOLD domain-containing protein; Involved in vesicular protein trafficking. Mainly functions in the early secretory pathway. Thought to act as cargo receptor at the lumenal side for incorporation of secretory cargo molecules into transport vesicles and to be involved in vesicle coat formation at the cytoplasmic side (By similarity) (212 aa); **SCORE:** 0.920

AKR2: ankyrin repeat-containing protein 2; Seems to be involved in the regulation of hydrogen peroxide levels during biotic and abiotic stresses by optimizing the ascorbate peroxidase 3 (APX3) hydrogen peroxide-degrading activity. This regulation might be monitored by GRF6 (350 aa)

APX3 L-ascorbate peroxidase; Plays a key role in hydrogen peroxide removal (By similarity) (287 aa); **SCORE:** 0.997

OEP7 outer envelope membrane protein 7 (64 aa); **SCORE:**0.996

TOC33 translocase of chloroplast 33; GTPase involved in protein precursor import into chloroplasts. Seems to recognize chloroplast-destined precursor proteins and regulate their presentation to the translocation channel through GTP hydrolysis. Binds GTP, GDP, XTP, but not ATP. Probably specialized in the import of nuclear encoded photosynthetic preproteins from the cytoplasm to the chloroplast, especially during early development stages (297 aa); **SCORE:** 0.989

TOC34 translocase of chloroplast 34; GTPase involved in protein precursor import into chloroplasts. Seems to recognize chloroplast-destined precursor proteins and regulate their presentation to the translocation channel through GTP hydrolysis. Probably specialized in the import of nuclear encoded non-photosynthetic preproteins from the cytoplasm to the chloroplast (313 aa); **SCORE:** 0.988

TOC64-III translocon at the outer membrane of chloroplasts 64-III; Chaperone receptor mediating Hsp90-dependent protein targeting to chloroplasts. Bi-functional preprotein receptor acting on both sides of the membrane. Not essential for an efficient import of pre-proteins into plastids (589 aa); **SCORE:** 0.946

CBR cytochrome-b5 reductase; Reductase transfering electrons from NADH to cytochrome b5. Required for the NADH-dependent electron transfer involved in the desaturation and hydroxylation of fatty acids and in the desaturation of sterol precursors. No activity with NADPH as electron donor (281 aa); **SCORE:**0.939

CB5-B cytochrome B5 isoform B; Membrane bound hemoprotein which function as an electron carrier for several membrane bound oxygenases, including fatty acid desaturases (134 aa); **SCORE:** 0.931

APX5 L-ascorbate peroxidase; Plays a key role in hydrogen peroxide removal (By similarity) (279 aa); **SCORE:** 0.930

AT1G07400 class I heat shock protein (157 aa); **SCORE:**0.866

HSP18.2 heat shock protein 18.2 (161 aa); **SCORE:** 0.795

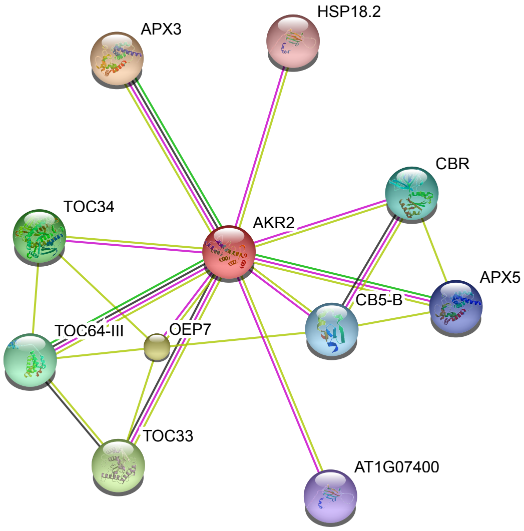

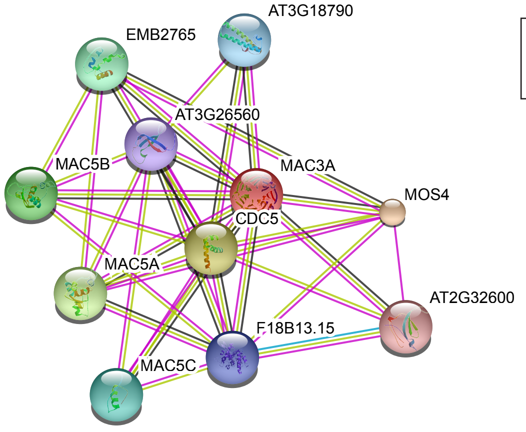

MAC3A MOS4-associated complex 3A; Component of the MAC complex that probably regulates defense responses through transcriptional control and thereby is essential for plant innate immunity. Functions as U-box E3 ubiquitin-protein ligase (By similarity). May also serve as a support for spliceosome binding and activity (By similarity) (523 aa)

MOS4 Modifier of snc1,4; Component of the MAC complex that probably regulates defense responses through transcriptional control and thereby is essential for plant innate immunity. Involved in mRNA splicing (By similarity) (253 aa); **SCORE:** 0.999

CDC5 cell division cycle 5; Component of the MAC complex that probably regulates defense responses through transcriptional control and thereby is essential for plant innate immunity. Possesses a sequence specific DNA sequence 'CTCAGCG' binding activity. Involved in mRNA splicing and cell cycle control (By similarity) (844 aa); **SCORE:** 0.999
MAC5A MOS4-associated complex subunit 5A (481 aa); **SCORE:** 0.997
MAC5B MOS4-associated complex subunit 5B (483 aa); **SCORE:** 0.997
EMB2765 EMBRYO DEFECTIVE 2765 (1509 aa); **SCORE:** 0.993
MAC5C MOS4-associated complex subunit 5C (363 aa); **SCORE:** 0.993
AT3G18790 pre-mRNA-splicing factor ISY1 (300 aa); **SCORE:** 0.992
F18B13.15 ABNORMAL SUSPENSOR 2 (2359 aa); **SCORE:** 0.991
AT3G26560 ATP-dependent RNA helicase DHX8/PRP22; Could act late in the splicing of pre-mRNA and mediate the release of the spliced mRNA from spliceosomes (By similarity) (1168 aa); **SCORE:** 0.986
AT2G32600 splicing factor 3A subunit 2 (277 aa); **SCORE:** 0.984

PDIL2-1, Protein disulfide-isomerase like 2-1, also referred to as UNE5 UNFERTILIZED EMBRYO SAC 5; Protein disulfide isomerase that may be required for proper pollen development, ovule fertilization and embryo development (361 aa)

CRT1b calreticulin-2; Molecular calcium-binding chaperone promoting folding, oligomeric assembly and quality control in the ER via the calreticulin/calnexin cycle. This lectin may interact transiently with almost all of the monoglucosylated glycoproteins that are synthesized in the ER (By similarity) (424 aa)**; SCORE:** 0.991
CNX1 calnexin 1; Calcium-binding protein that interacts with newly synthesized glycoproteins in the endoplasmic reticulum. It may act in assisting protein assembly and/or in the retention within the ER of unassembled protein subunits. It seems to play a major role in the quality control apparatus of the ER by the retention of incorrectly folded proteins (By similarity) (530 aa)**; SCORE:** 0.989
GLU2 glutamate synthase 2; May play a role in primary nitrogen assimilation in roots. Could supply a constitutive level of glutamate to maintain a basal level of protein synthesis (1629 aa)**; SCORE:** 0.978
GLT1 glutamate synthase 1 [NADH]; Involved in glutamate biosynthesis. Required for non- photorespiratory ammonium assimilation. Probably involved in primary ammonium assimilation in roots (2208 aa)**; SCORE:** 0.978
GLU1 glutamate synthase 1; Involved in glutamate biosynthesis in leaf. Required for the reassimilation of ammonium ions generated during photorespiration (1648 aa)**; SCORE:** 0.976
SHD SHEPHERD; May have a molecular chaperone role in the processing of secreted materials. Required for shoot apical meristem (SAM), root apical meristem (RAM) and floral meristem (FM) formation, probably by regulating the folding of CLAVATA proteins (CLVs). Also involved in pollen tube elongation (823 aa)**; SCORE:** 0.973
PDIL1-1 PDI-like 1-1; Protein disulfide isomerase that associates with RD21A protease for trafficking from the ER through the Golgi to lytic and protein storage vacuoles of endothelial cells in developing seeds. Regulates the timing of programmed cell death (PCD) of the endothelial cells by chaperoning and inhibiting cysteine proteases during their trafficking to vacuoles (501 aa)**; SCORE:** 0.972
PDIL1-2 PDI-like 1-2; Acts as a protein-folding catalyst that interacts with nascent polypeptides to catalyze the formation, isomerization, and reduction or oxidation of disulfide bonds (By similarity) (508 aa)**; SCORE:** 0.965
ERO2 endoplasmic oxidoreductin-2; Essential oxidoreductase that oxidizes proteins in the endoplasmic reticulum to produce disulfide bonds. Acts by oxidizing directly PDI isomerase through a direct disulfide exchange. Does not act as a direct oxidant of folding substrate, but relies on PDI to transfer oxidizing equivalent. Does not oxidize all PDI related proteins, suggesting that it can discriminate between PDI and related proteins. Its reoxidation probably involves electron transfer to molecular oxygen via FAD. Acts independently of glutathione. May be responsible for a significant proport [...] (479 aa)**; SCORE:** 0.949
ERO1 endoplasmic oxidoreductin-1; Essential oxidoreductase that oxidizes proteins in the endoplasmic reticulum to produce disulfide bonds. Acts by oxidizing directly PDI isomerase through a direct disulfide exchange. Does not act as a direct oxidant of folding substrate, but relies on PDI to transfer oxidizing equivalent. Does not oxidize all PDI related proteins, suggesting that it can discriminate between PDI and related proteins. Its reoxidation probably involves electron transfer to molecular oxygen via FAD. Acts independently of glutathione. May be responsible for a significant proport [...] (469 aa)**; SCORE:** 0.949

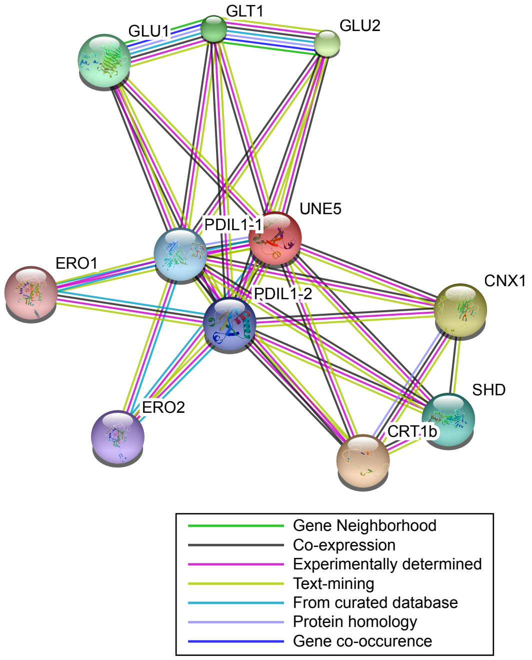

AT3G09440 protein heat shock protein 70-3; In cooperation with other chaperones, Hsp70s stabilize preexistent proteins against aggregation and mediate the folding of newly translated polypeptides in the cytosol as well as within organelles. These chaperones participate in all these processes through their ability to recognize nonnative conformations of other proteins. They bind extended peptide segments with a net hydrophobic character exposed by polypeptides during translation and membrane translocation, or following stress-induced damage (By similarity) (649 aa)

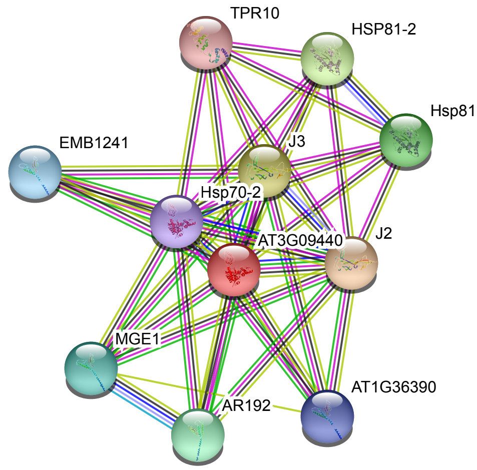

J2 DNAJ homologue 2; Have a continuous role in plant development probably in the structural organization of compartments (By similarity) (419 aa); **Score:** 0.998
J3 chaperone protein dnaJ 3; Have a continuous role in plant development probably in the structural organization of compartments (By similarity) (420 aa); **Score:**0.996
HSP81-2 heat shock protein 81-2; Molecular chaperone involved in RPM1-mediated resistance. Component of the RPM1/RAR1/SGT1 complex. May stabilize RPM1 and protect it from SGT1-mediated degradation. Associates with RAR1 which may function as co-chaperone. Possesses ATPase activity (728 aa); **Score:**0.994
Hsp81.4 HEAT SHOCK PROTEIN 81.4; Molecular chaperone. Due to its association with certain proteins such as hormone receptors and some classes of kinases, it is implicated in signal transduction and development. Has ATPase activity (By similarity) (699 aa); **Score:**0.994
AR192 molecular chaperone GrpE; Essential component of the PAM complex, a complex required for the translocation of transit peptide-containing proteins from the inner membrane into the mitochondrial matrix in an ATP-dependent manner (By similarity) (327 aa); **Score:**0.994
MGE1 mitochondrial GrpE 1; Essential component of the PAM complex, a complex required for the translocation of transit peptide-containing proteins from the inner membrane into the mitochondrial matrix in an ATP-dependent manner (By similarity) (302 aa); **Score:** 0.993
EMB1241 embryo defective 1241; Essential component of the PAM complex, a complex required for the translocation of transit peptide-containing proteins from the inner membrane into the mitochondrial matrix in an ATP-dependent manner (By similarity) (326 aa); **Score:** 0.993
AT1G36390 co-chaperone grpE-like protein; Essential component of the PAM complex, a complex required for the translocation of transit peptide-containing proteins from the inner membrane into the mitochondrial matrix in an ATP-dependent manner (By similarity) (279 aa); **Score:** 0.993
Hsp70-2 heat shock protein 70; Component of the Mediator complex, a coactivator involved in the regulated transcription of nearly all RNA polymerase II-dependent genes. Mediator functions as a bridge to convey information from gene-specific regulatory proteins to the basal RNA polymerase II transcription machinery. The Mediator complex, having a compact conformation in its free form, is recruited to promoters by direct interactions with regulatory proteins and serves for the assembly of a functional preinitiation complex with RNA polymerase II and the general transcription factors (By similarity) (653 aa); **Score:** 0.987
TPR10 tetratricopeptide repeat 10 (680 aa); **Score:**0.986
